# Large-Scale Biomedical Relation Extraction Across Diverse Relation Types: Model Development and Usability Study on COVID-19

**DOI:** 10.2196/48115

**Published:** 2023-09-20

**Authors:** Zeyu Zhang, Meng Fang, Rebecca Wu, Hui Zong, Honglian Huang, Yuantao Tong, Yujia Xie, Shiyang Cheng, Ziyi Wei, M James C Crabbe, Xiaoyan Zhang, Ying Wang

**Affiliations:** 1 Research Center for Translational Medicine Shanghai East Hospital, School of Life Sciences and Technology Tongji University Shanghai China; 2 Department of Clinical Laboratory Medicine Center Yueyang Hospital of Integrated Traditional Chinese and Western Medicine Shanghai University of Traditional Chinese Medicine Shanghai China; 3 Department of Laboratory Medicine Shanghai Eastern Hepatobiliary Surgery Hospital Shanghai China; 4 University of California, Berkeley Berkeley, CA United States; 5 Institutes for Systems Genetics Frontiers Science Center for Disease-Related Molecular Network, West China Hospital Sichuan University Chengdu China; 6 Wolfson College Oxford University Oxford United Kingdom; 7 Institute of Biomedical and Environmental Science & Technology University of Bedfordshire Luton United Kingdom; 8 School of Life Sciences Shanxi University Taiyuan China

**Keywords:** biomedical text mining, biomedical relation extraction, pretrained language model, task-adaptive pretraining, knowledge graph, knowledge discovery, clinical drug path, COVID-19

## Abstract

**Background:**

Biomedical relation extraction (RE) is of great importance for researchers to conduct systematic biomedical studies. It not only helps knowledge mining, such as knowledge graphs and novel knowledge discovery, but also promotes translational applications, such as clinical diagnosis, decision-making, and precision medicine. However, the relations between biomedical entities are complex and diverse, and comprehensive biomedical RE is not yet well established.

**Objective:**

We aimed to investigate and improve large-scale RE with diverse relation types and conduct usability studies with application scenarios to optimize biomedical text mining.

**Methods:**

Data sets containing 125 relation types with different entity semantic levels were constructed to evaluate the impact of entity semantic information on RE, and performance analysis was conducted on different model architectures and domain models. This study also proposed a continued pretraining strategy and integrated models with scripts into a tool. Furthermore, this study applied RE to the COVID-19 corpus with article topics and application scenarios of clinical interest to assess and demonstrate its biological interpretability and usability.

**Results:**

The performance analysis revealed that RE achieves the best performance when the detailed semantic type is provided. For a single model, PubMedBERT with continued pretraining performed the best, with an F1-score of 0.8998. Usability studies on COVID-19 demonstrated the interpretability and usability of RE, and a relation graph database was constructed, which was used to reveal existing and novel drug paths with edge explanations. The models (including pretrained and fine-tuned models), integrated tool (Docker), and generated data (including the COVID-19 relation graph database and drug paths) have been made publicly available to the biomedical text mining community and clinical researchers.

**Conclusions:**

This study provided a comprehensive analysis of RE with diverse relation types. Optimized RE models and tools for diverse relation types were developed, which can be widely used in biomedical text mining. Our usability studies provided a proof-of-concept demonstration of how large-scale RE can be leveraged to facilitate novel research.

## Introduction

### Background

With the rapid research and development of new biotechnologies, a vast amount of biomedical literature has been published, and biomedical text mining has proved to be invaluable for analyzing the literature, especially for some hot topics that have received much attention from scientific research, such as drug development and public health events. For instance, the COVID-19 pandemic generated a large number of research articles (over 368,000 as of August 3, 2023), making it a priority for the biomedical text mining research community to extract structured knowledge from the corpus of information. The infeasibility of manually reviewing large-scale literature leads to knowledge bottlenecks that contribute to the duplication of research efforts and inefficiency in the development of treatment strategies. The development of better knowledge mining technologies will enable researchers to access relevant knowledge more efficiently and accurately.

Biomedical natural language processing (NLP) techniques are used to extract useful information from biomedical text, such as scientific literature and electronic health records, and these techniques have been extensively employed to extract and retrieve structured information from massive article collections [1]. A critical part of biomedical NLP is relation extraction (RE), which associates a given sentence or text containing entity information with a relation type based on characteristics (entities, context, and semantic features) under predefined categories. RE can be used to implement many application scenarios, such as a structured search and knowledge summarization, and is also the key component for building knowledge graphs (KGs), a powerful way to represent and integrate large-scale textual data to generate new insights [2].

### Related Work

Biomedical RE can be performed with a variety of algorithms and data sets. Previous studies adopted computational methods to extract relations, including co-occurrence–based methods, pattern-based methods [3], rule-based methods, feature-based methods [4], and kernel-based methods [5]. For instance, SemRep is a notable rule-based RE tool developed by the National Library of Medicine that can extract semantic relations from sentences in biomedical text [6,7]. Deep neural network–based methods have been shown to achieve better results in various NLP tasks and to have good performance in automatic feature learning [8]. Recent advances in text mining focus on pretrained language models, and many studies have shown that pretrained language models have achieved state-of-the-art NLP methodologies for biomedical text mining [9]. Data sets also play a critical role in deep learning–based RE models, as many RE data sets and models have been built. However, these tasks are mainly focused on the general NLP domain, and the data sets are usually taken from public domain articles, such as Wikipedia [10]. Most existing RE data sets in the biomedical domain, such as GDR [11], are limited in the amount of data and diversity of relations due to labor-intensive manual annotation. BioRel is a large-scale RE data set encompassing 125 biomedical relations via knowledge database utilization and distant supervision [12]. It could facilitate the development of deep learning methods in the extraction of biomedical relations. Although BioRel provides a large-scale data set and has an abundance of biomedical relation types, as far as we know, this data set has not been explored at the entity type level, especially with regard to the influence of the abundance of entity types on RE performance. Moreover, to our knowledge, no research has applied the model with continued pretraining to biomedical RE to build tools and apply them to practical biomedical problems.

### Objective

**Figure 1:**
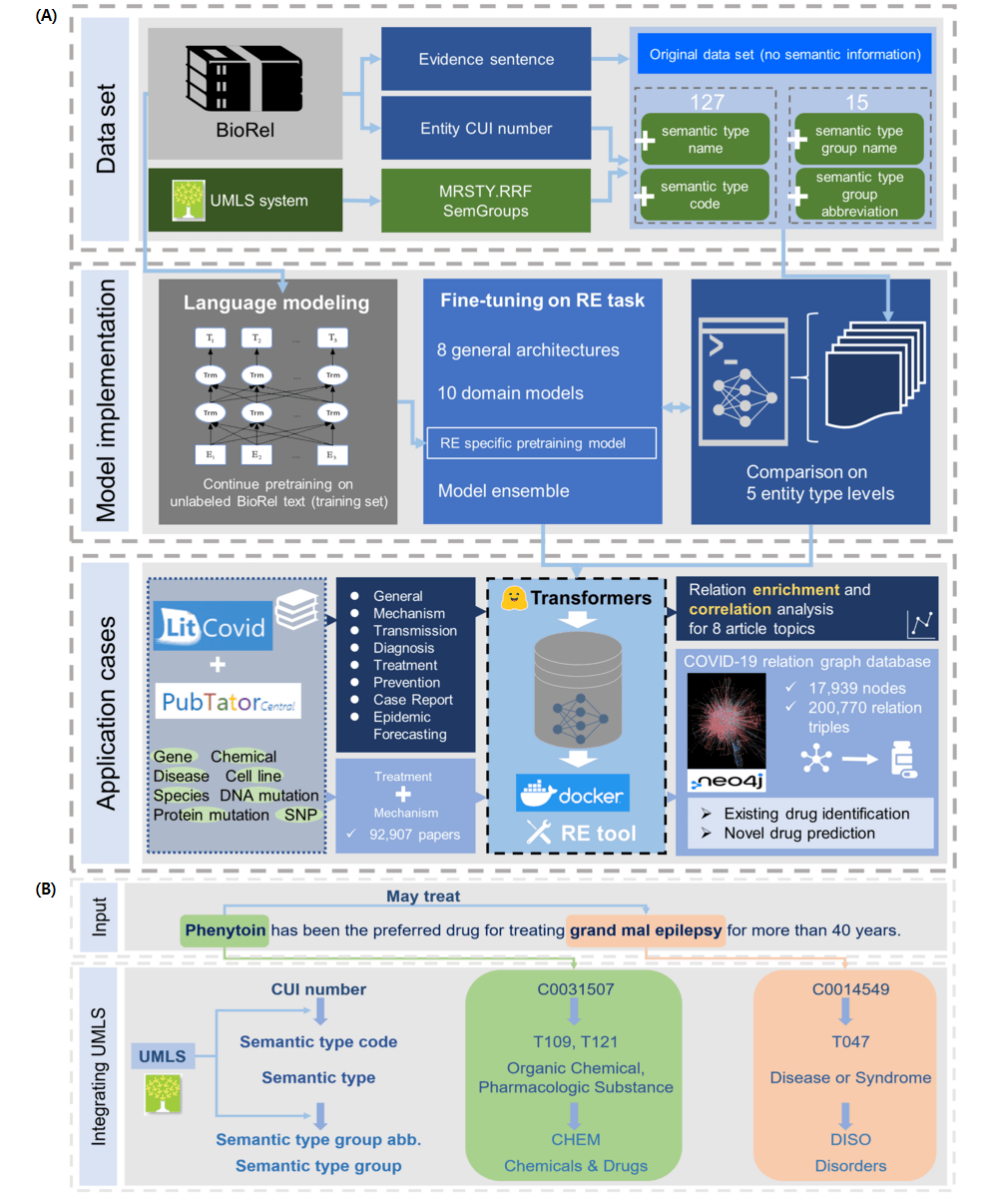
Flowchart of the relation extraction (RE) study and data set construction. (A) Overview flow diagram of 3 main modules in this study. (B) Demonstration of integrating Unified Medical Language System (UMLS) entity information into the data set for a sentence with entity annotation. CUI: Concept Unique Identifier.

In order to investigate and improve large-scale RE with diverse relation types in biomedical text mining, we conducted a comprehensive study, including a modeling study, tool development, and a usability study with COVID-19 literature. As shown in Figure 1A, we first integrated the Unified Medical Language System (UMLS) [13], one of the most widely used knowledge resources in biomedical NLP, with the BioRel data set to provide richer entity information, generating 4 data sets with different semantic levels of entities. Next, we conducted RE model implementations and evaluated the impact of different levels of semantic information on RE performance. We also compared a variety of pretrained model architectures and domain models, including 8 general pretrained model architectures and 10 domain models, and adopted continued pretraining strategies and ensemble modeling to improve RE performance. The fine-tuned models were integrated into an RE tool as a Docker container. In addition, we investigated biological interpretability and performed multiple application cases on the COVID-19 corpus, including relation enrichment/correlation among topics, relation graph database construction with existing drug identification and novel drug path prediction, non-long/long COVID drug retrieval, and coronavirus-specific relation triple prediction. Our results provide novel insights into biomedical RE in large-scale literature text mining for future studies. The RE task-specific pretrained and fine-tuned models are publicly available at Hugging Face Hub [14-18], and the RE tool is publicly available at Docker Hub [19].

## Methods

### Data Collection and Processing

Many biomedical RE data sets focus on some specific entities and relations, and the data sizes are relatively small. To cover a wide range of biomedical relations and facilitate pretrained model strengths on large data sets, we conducted RE model experiments on BioRel [12], a large-scale data set that includes more than 763,000 sentences (534,000 in the training set) and 69,000 entities. It consists of 125 relations (including “not a relation”) that cover most biomedical relation types, including treatment, side effects, and mechanisms. It also contains relation directions like A “may treat” B, and B “may be treated by” A. Examples of relation types and sentences are shown in Multimedia Appendix 1. The data set involves multiple entity types, so we matched these entities to the UMLS database using a Concept Unique Identifier (CUI) number to obtain entity semantic information, generating 4 data sets with different entity type information levels. Specifically, we first downloaded UMLS metathesaurus data in rich release format (RRF), which includes all semantic type RRF information with a CUI number, and extracted 127 semantic type names and unique identifiers of semantic types (semantic type codes) by CUI number. Then, we downloaded semantic group data from the UMLS semantic network and extracted 15 semantic type group names and unique identifiers of semantic type groups (semantic type group abbreviations) by semantic type code, which involved the other 2 data sets. Figure 1B shows an example of integrating UMLS entity information to generate 4 data sets for a sentence with a CUI number. So far, we have the following 5 data sets with different entity levels, including the original data set: semantic type name, semantic type code, semantic type group name, semantic type group abbreviation, and no entity type.

In the RE application case, the COVID-19 literature corpus was retrieved from the LitCovid database [20] and the CoronaCentral database [21]. LitCovid is a database (daily updated and curated) for tracking COVID-19 scientific literature that provides article topic categories through manual assignment. The categories are “general information,” “mechanism,” “transmission,” “diagnosis,” “treatment,” “prevention,” “case report,” and “epidemic forecasting.” LitCovid also provides a long COVID collection comprising articles that investigated the persistent, long-term, or delayed symptoms of COVID-19, as well as complications from its treatment at least 4 weeks after the onset of symptoms. We downloaded the LitCovid corpus with PubTator [22,23] detection, which provides automatic entity annotation of 6 biomedical concepts (genes, chemicals, diseases, cell lines, species, and variations). Given the LitCovid categories, we mainly focused on treatment and mechanism literature to extract existing and novel drug therapy knowledge by relation graph database construction. CoronaCentral is a resource providing coronavirus papers containing coronavirus-specific entity recognitions, such as “prevention methods,” “risk factors,” “transmission types,” and “vaccine types.” The coronavirus articles with virus-specific mentions were used to obtain a more specific biomedical association and improve the research community’s understanding of the coronavirus.

### Ethical Considerations

This work does not involve any ethical or moral issues. The data used in this study are publicly available dataset and literature data. This study did not meet the criteria for human subject research and a review by an institutional review board was not required.

### Language Model Implementation

To evaluate the impact of different levels of semantic information on the performance of RE, we compared the performance of the 4 generated data sets and the original data set based on the same language model. Likewise, we conducted performance analysis on different pretrained model architectures and domain models based on the same data set for semantic level (semantic type name). General pretrained model architectures include ALBERT [24], BERT [25,26], DeBERTa [27], ELECTRA [28], ERNIE [29], LayoutLM [30], RoBERTa [31], and XLNet [32]. For the biomedical domain, we adopted 10 popular models with different pretraining corpus and parameter settings: BioM-ALBERT [33], BioM-ELECTRA [33], PubMedELECTRA [34], BioMed-RoBERTa [35], RoBERTa-PubMed [36], BioBERT [9], SciBERT [37], BioClinicalBERT [38], BlueBERT [39], and PubMedBERT [40].

Moreover, inspired by Gururangan et al [35] (improving performance through task-adaptive pretraining on unlabeled data was found to be effective even after undergoing domain-adaptive pretraining), we proposed a RE-specific continued pretraining strategy to specialize a model and achieve better performance. We adopted the best-performing model to continue pretraining on the unlabeled text (raw text) of the BioRel training set. Continued pretraining was based on masked language modeling (MLM) with the sliding window technique to predict masked tokens in sequence to obtain good contextual understanding, and then, we fine-tuned the RE-specific pretrained model on the labeled training set to evaluate whether performance improved. We also applied an ensemble model integrating the top-performing models to improve the RE performance in this task. The SoftMax function was used to determine the probability that each single model would correctly predict the labels of the relations for input text in the ensemble layer after obtaining the logits of each single model. We employed the ensemble model to produce the final results by using the concept of weighted average to accept the matrix and labels of the submodel prediction scores as input.

Based on the idea of R-BERT [41], tokens of the model input with different entity levels and special separate tokens on both sides were transformed into numerical vectors containing semantic features, which were presented as token embeddings of the model input representation. For the continued pretraining configuring, the ratio of tokens to mask for MLM loss was 0.15 and the fraction for stride in the sliding window was 0.8. We ran 100 epochs with a batch size of 150. For the fine-tuning hyperparameter, the learning rate was set at 2×10^−5^ and the batch size was set at 64, and we ran 5 epochs for each model. Due to the fact that most sentences in the data set were less than 128 words in length, the maximum length of input was set at 128. Finally, labels of relations were output using a fully connected layer. The cross-entropy loss was used as the loss function, and AdamW was used as the optimizer. These language models were implemented with PyTorch (version 1.12.0), Hugging Face Transformers (version 4.26.1) [42], and open source pretraining parameters. Regarding hardware, we conducted pretraining on an NVIDIA GeForce RTX 3090 (24 GB) graphics card. In the fine-tuning phase, we carried out experiments on 2 NVIDIA Tesla P100 (12 GB) systems.

### Evaluation Metrics

The models were fine-tuned on the training set and evaluated on the testing set. The testing set included a total of 114,515 samples and covered 125 relation types, and the proportion of each relation type was consistent with the training set. To measure the impact of entity level on biomedical RE tasks and evaluate the performance of language models, we used deep learning metrics that are often adopted: precision, recall, and F1-score. The benchmark score for this task, which combined precision and recall, was the weighted average F1-score. The formulae are presented in Multimedia Appendix 2. These metrics were aggregated across 125 relation types in weighted average levels to compare the overall performance. Precision-recall curves were drawn for 5 entity levels and all language models. 

### Usability Study With the COVID-19 Corpus

To demonstrate the biological significance and interpretability of the RE application, we conducted experiments to examine relation type enrichment and correlation within different article topics. Equal numbers of articles covering 8 topics from LitCovid were filtered out separately (downloaded on March 7, 2023), and then, we detected relations from these 8 sets to compare relation enrichment between topics. The correlation index between topics by diversity and amount of relation was calculated to illustrate the strength of the correlation between every 2 topics. Principal component analysis (PCA) was also performed to display the distance between the 8 topics based on relation type. 

To understand the clinical treatment and drug discovery for COVID-19, based on the KG concept, we retrieved “treatment” and “mechanism” research from LitCovid to build a relation graph database. Sentences with entities were used in RE prediction, and the results were encoded in triple format (ie, head node–relation–tail node), producing metadata regarding the nodes and their relational connections, as well as the correlating origins and context. The triple data mainly contained 2 entities and their relation, including the direction of the relation. We converted these triple data and imported them into Neo4j, a popular graph database management system, using custom Cypher scripts.

Moreover, we used the relation graph database to identify existing drugs and discover novel drug candidates. For existing drugs, we defined 3 conditions to identify drugs with more plausible associations with COVID-19, which also helped us to visualize the paths associated with the drug-disease. To discover potential drugs not directly connected to COVID-19, we adopted the drug paths defined by Sosa et al [43]. They used the Global Network of Biomedical Relationships (GNBR) [44], a large KG, to predict new drug paths for rare diseases, and they defined three 4-node paths connecting the drug-disease. Within path a, the medication uses the same genetic process to treat a different condition. Within path b, the medication addresses a condition that is treated similarly to the condition of interest. Within path c, the medication addresses the condition through 2 connected genes. We also added additional qualifications on the 3 drug paths based on the original pattern to find more plausible nodes and edges. The results were ranked by the Adamic Adar score [45]. This algorithm was used to compute the closeness of nodes based on their shared neighbors. The calculation of the Adamic Adar score is shown in Multimedia Appendix 2.

## Results

### Impact of Entity Information Level and Language Model Implementation

**Figure 2:**
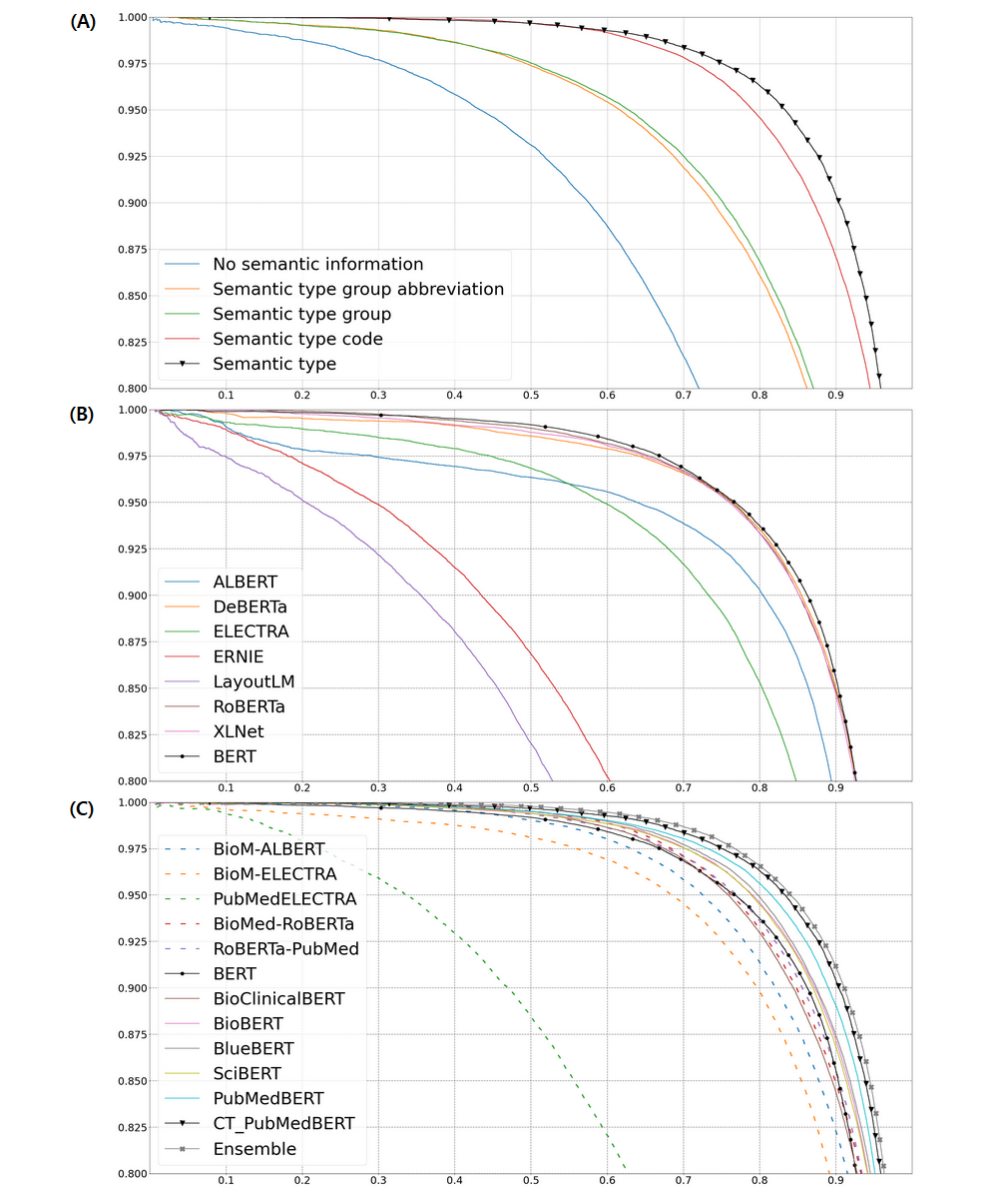
Precision-recall curves (x-axis: recall, y-axis: precision). (A) The performance comparison of 5 entity levels. (B) The performance comparison of 8 pretrained model architectures. (C) The performance comparison of 10 domain models, CT_PubMedBERT, and the model ensemble (BERT included for comparison). BERT-based domain models are represented by solid lines, and nonBERT-based domain models are represented by dashed lines.

Data sets with 5 different entity levels were used for experiments, and they were fine-tuned with the same pretrained model (PubMedBERT with continued pretraining) for comparison. The precision-recall curves are shown in Figure 2A. The performance of RE was the best when using semantic name (F1-score=0.8998) and semantic name code (F1-score=0.8817), the next best when using semantic type group (F1-score=0.8279) and semantic type group abbreviation (F1-score=0.8230), and the worst when using the original data set that had no entity information (F1-score=0.7440). The performance values for semantic name were slightly higher than for semantic name code, and were slightly higher for semantic type group than for semantic type group abbreviation. The results showed that the detailed semantic name can provide more semantic information than the abbreviation and code name to improve the performance of RE. Multimedia Appendix 3 shows the F1-score difference of each relation type for the 4 entity information levels compared to the no semantic type. It can be seen that “semantic type group abbreviation” had 104 relation types with improvement, 13 with no change, and 8 with a decrease; “semantic type group” had 112 relation types with improvement, 10 with no change, and 3 with a decrease; “semantic type code” had 115 relation types with improvement, 7 with no change, and 3 with a decrease; and “semantic type” had 125 relation types with improvement. Regarding the specific performance of each relation type, there were some relation types with substantial performance improvement at all 4 levels, such as “gene encodes gene product” and “process involves gene,” as these relation types tended to concentrate on certain fixed combinations. Similarly, when using “semantic type” and “semantic type code,” some relation types were further enhanced compared to “semantic type group” and “semantic type group abbreviation,” such as “associated with malfunction of gene product” and “has physiologic effect,” indicating that these relation types were more focused on the combination of entity types. In addition to the above-mentioned relation types that were affected by entity type combination, there were also relation types whose performances were affected by the semantic features behind the entity type information, resulting in “semantic type group” and “semantic type” performing better than “semantic type group abbreviation” and “semantic type code,” respectively, such as “disease excludes primary anatomic site” and “is abnormal cell of disease,” indicating that the full texts of “semantic type group” and “semantic type” were transformed into numerical vectors containing semantic features during tokenization, such that the model input representation had richer token embeddings, which was helpful for the model to identify the relation type. Therefore, entity semantic information helps RE from not only the combination of entity types, but also its semantic features in token embeddings.

We implemented a variety of pretrained model architectures and domain models. The results are shown in Figure 2B, Figure 2C, and Table 1. We used the precision-recall curve to visualize performance comparison, and the precision, recall, and F1-score to evaluate the performance of each pretrained model. For general model architecture, the performance of BERT was slightly better than that of DeBERTa, RoBERTa, and XLNet, achieving an F1-score of 0.8790. For domain models, PubMedBERT with our continued pretraining (CT_PubMedBERT) achieved the best results for a single language model, with a weighted average precision of 0.8998, recall of 0.9010, and F1-score of 0.8998. The ensemble model integrating BioBERT, BlueBERT, and CT_PubMedBERT with continued pretraining (the 3 best-performing single models) achieved the best overall results with an average precision of 0.9026, recall of 0.9046, and F1-score of 0.9028.

**Table 1:** Precision, recall, and F1-score of different architectures and models.

Architecture and model		Precision	Recall	F1-score
**ALBERT**				
	ALBERT	0.852	0.8566	0.8533
	BioM-ALBERT	0.8611	0.8625	0.8577
**BERT**				
	BERT	0.8784	0.881	0.879
	BioClinicalBERT	0.8675	0.8718	0.8684
	BioBERT	0.8836	0.8857	0.8838
	SciBERT	0.8808	0.8834	0.8815
	BlueBERT	0.8833	0.8865	0.8838
	PubMedBERT	0.892	0.894	0.8925
	CT_PubMedBERT	0.8998	0.901	0.8998
	Ensemble^a^	0.9026	0.9046	0.9028
**DeBERTa**				
	DeBERTa	0.8757	0.8775	0.8763
**ELECTRA**				
	ELECTRA	0.8231	0.8249	0.8236
	BioM-ELECTRA	0.848	0.8508	0.8487
	PubMedELECTRA	0.6904	0.7104	0.6916
**ERNIE**				
	ERNIE	0.6753	0.7007	0.6798
**LayoutLM**				
	LayoutLM	0.6287	0.6645	0.6311
**RoBERTa**				
	RoBERTa	0.8736	0.8752	0.874
	BioMed-RoBERTa	0.8685	0.874	0.8696
	RoBERTa-PubMed	0.8752	0.878	0.8758
**XLNet**				
	XLNet	0.8749	0.8762	0.8754

^a^The ensemble model integrated BioBERT, BlueBERT, and CT_PubMedBERT with continued pretraining.

The F1-scores for each relation label in CT_PubMedBERT are shown in Multimedia Appendix 4. There were 48 relation labels (87,369/114,565, 76.3% of the total testing set) that had F1-scores greater than 90%, and the categories that performed the best were “biological process involves gene product,” “chemical or drug initiates biological process,” “chemotherapy regimen has component,” “gene encodes gene product,” “gene product encoded by gene,” “gene product has organism source,” “gene product plays role in biological process,” “is component of chemotherapy regimen,” “is organism source of gene product,” and “organism has gene.” These categories all demonstrated a precision, recall, and F1-score of 99%. Multimedia Appendix 5 shows the F1-score change of CT_PubMedBERT compared with PubMedBERT for each relation type. We can see that CT_PubMedBERT improved most relation types (improved 88 relation types, caused no change in 6 relation types, and decreased 31 relation types). Specifically, CT_PubMedBERT showed improvement over PubMedBERT for relation types with low performance owing to lack of data (the proportion of the relation type in the data set reflects its probability of occurrence in real scenarios), and 13 of the 15 relation types with F1-scores of less than 0.5 showed improvement (1 had no change and 1 decreased), indicating that the task-specific language modeling of CT_PubMedBERT has advantages for identifying minority relation types.

We set the epoch to 100 to improve visualization of the F1-score trend and training step loss for our large data set and complex relation types. As shown in Multimedia Appendix 6, the F1-score increased continuously, and the loss continued to decrease even after 100 epochs. The focal loss in the training set converged more slowly, but still fluctuated and decreased slightly. The model did not fit the large data set perfectly even after 100 epochs. Nonetheless, in terms of running time and performance evaluation, the results were satisfactory after just 5 epochs.

Owing to complicated and heavy deep learning dependencies that are difficult to install and launch, we compiled a Docker image with all the relevant frameworks for this RE model. It contained all the required libraries, packages, and files, saving time for framework installation. The usage tutorials and documentation for the interactive Jupyter Notebook are provided on the Docker hub [19], and the RE tool is publicly available as a Docker container online. We also uploaded our continued pretraining model and its fine-tuned RE models to the Hugging Face Hub [14-18].

### Usability Analysis With COVID-19 Cases

**Figure 3:**
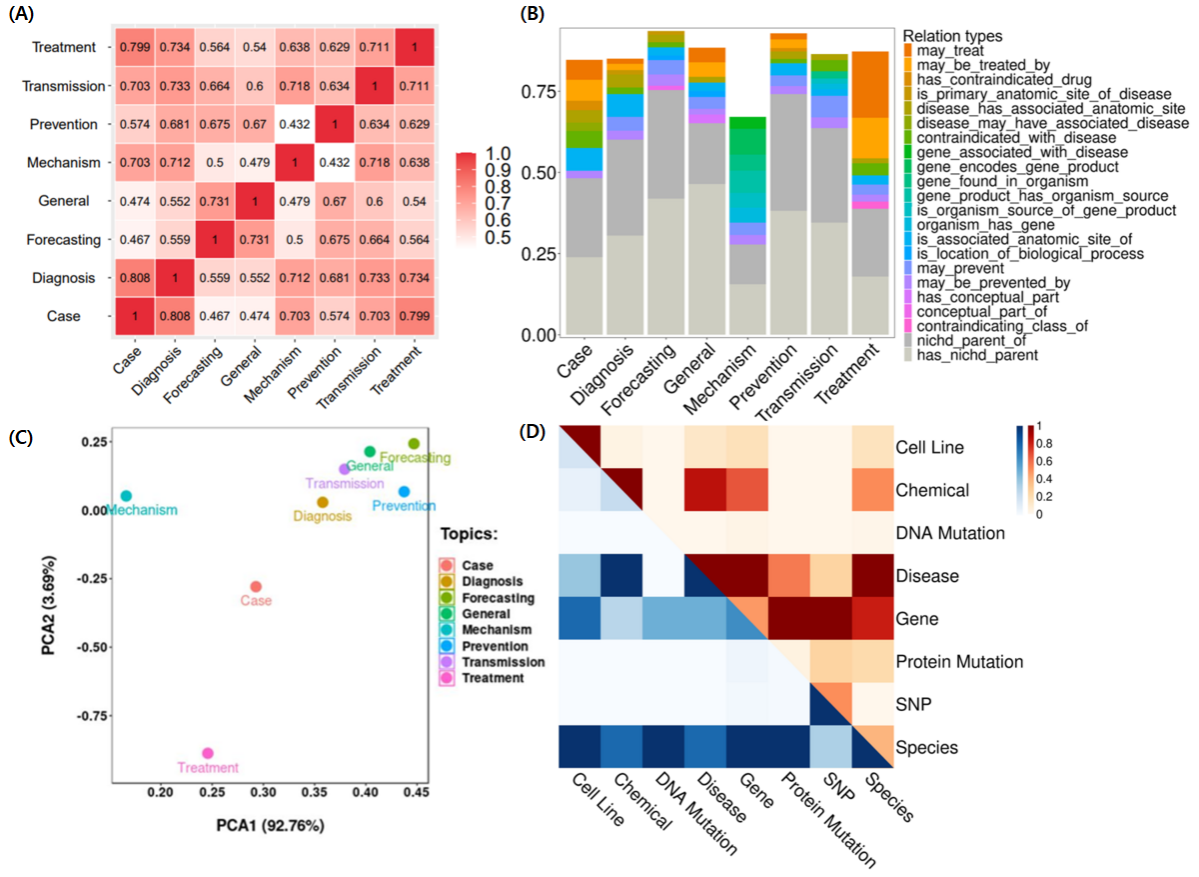
Visualization of topics and entities based on relation extraction (RE). (A) Pairwise correlation between 8 topics calculated by relations. (B) Proportion of the top 10 relations among 8 topics. (C) Principal component analysis (PCA) of 8 topics. (D) Strength of correlations between different types of entities calculated by RE. The top right part is calculated by the number of relations, and the bottom left part is calculated by the number of relation types. SNP: single-nucleotide polymorphism.

The biological significance of RE was evaluated by correlation and relation enrichment. Figure 3A shows the pairwise correlation between 8 topics calculated by relations, demonstrating the strength of the correlation between 2 topics from the perspective of RE. The correlations between “case report” and “diagnosis,” and “case report” and “treatment” were relatively high, while the correlations between “mechanism” and “prevention,” and “mechanism” and “general” were relatively low. Macroscopically, descriptions related to “diagnosis” and “treatment” of patients were often included in many “case report” articles, while “mechanism” research of viruses often investigated specific biological processes and gene activities, and it did not involve “general” descriptions and “prevention” information. Therefore, the correlation coefficient calculated from RE conformed to the actual content overlap between different topics. We also compared the proportions of the top 10 relation types in each of the 8 article types. Figure 3B shows that in the “treatment” topic, the proportion of treatment-related relation types was relatively high, while in the “mechanism” topic, the proportion of relation types related to gene function was relatively high, which shows that the RE model had biological significance. Specifically, Figure 3C shows the PCA of relation type for 8 topics. The distance between 2 topics represented their relation-based similarity, and articles involving “treatment,” “mechanism,” and “case report” had relatively topic-specific relation types compared with other topics. We also performed visualization of relation strength between different entity types by the number of relations and relation type, as shown in Figure 3D. “Gene,” “disease,” and “chemical” had strong RE associations with other entity types, as did “species,” owing to the inclusion of “SARS-CoV-2,” and these findings are in line with real scenarios.

We built a COVID-19 relation graph database composed of mechanism and treatment research. The abstracts were downloaded on June 12, 2023, and the RE pipeline was performed. A total of 1,849,915 relation triples were identified in 92,907 papers. After merging identical triples and removing “not a relation” triples, a total of 17,939 unique entities and 200,770 unique relation triples were imported into the Neo4j graph database (version 4.4.21), with node attributes containing entity names and normalized IDs, and edge attributes containing the relation type, the direction, the number of sources, and the mean and sum of the probabilities. The relation graph database constituted a coherent network and allowed entities and relations to be queried, browsed, and navigated. The visualization made it possible for us to view and analyze the network by filtering nodes or edges, and allowed us to explore paths from one piece of information to another. Moreover, it allowed us to add updated information and analyze its indications against the relation graph database. Multimedia Appendix 7 shows a demonstration of the relation graph database. The orange nodes are diseases, the brown nodes are genes, and the purple nodes are chemicals or drugs. Owing to the data sources, the causation relations from “treatment” papers predominantly reflected COVID-19 pathologies and therapies, and those from “mechanism” papers mainly reflected molecular interactions and biological processes. For example, as shown in Multimedia Appendix 7, we can visualize how topotecan acts as a potential drug through the relation graph database (topotecan reduces SARS-CoV-2–induced inflammation by affecting human topoisomerase 1 [46,47]). The importable data of the relation graph database for Neo4j are publicly available [48].

**Figure 4:**
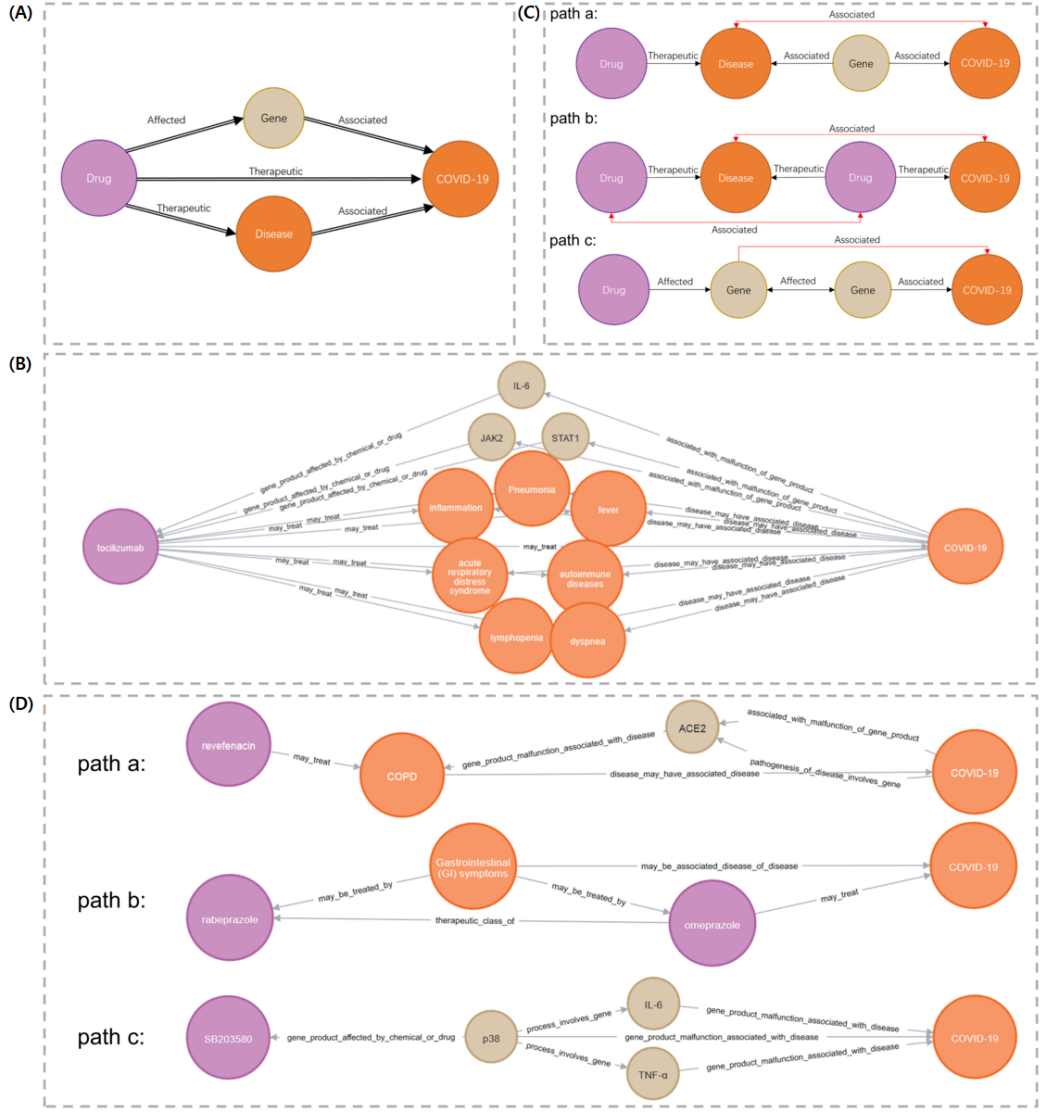
Existing and novel drug graphs. (A) Existing drug identification patterns. (B) Existing drug identification examples. (C) Novel potential drug prediction patterns. (D) Novel potential drug examples. ACE2: angiotensin converting enzyme 2; COPD: chronic obstructive pulmonary disease; IL: interleukin; JAK2: Janus kinase 2; STAT1: signal transducer and activator of transcription 1; TNFα: tumor necrosis factor-α.

The relation graph database was notable for its extensive coverage of drug-disease interactions, as well as the associated nodes of biological mechanisms linked to COVID-19. As shown in Figure 4A, to identify existing drugs having more plausible associations with COVID-19, 3 conditions must be met. First, the drug should be able to produce an effect on a gene associated with COVID-19. Second, the drug should be therapeutic for a disease associated with COVID-19. Third, the above relations should be identified in at least two different texts to ensure plausibility. The top 3 results, ordered by the Adamic Adar score, were tocilizumab, ritonavir, and baricitinib. The graph for tocilizumab (ranked first) is shown in Figure 4B. Tocilizumab mainly affects interleukin 6 (IL-6), Janus kinase 2 (JAK2), and signal transducer and activator of transcription 1 (STAT1) [49,50], while it has a therapeutic effect on COVID-19–related inflammation, dyspnea, and autoimmune diseases [51-53].

To discover novel potential drugs, we used the relation graph database to retrieve 3 drug paths. As shown in Figure 4C, the first node drug in paths a, b, and c is not directly connected to COVID-19 in the entire relation graph database but is connected to COVID-19 through 2 intermediate nodes. The black edges represent the interentity connections defined by Sosa et al [43], and the red edges are the edge qualifications we performed. In all 3 paths, the second node (disease or gene) must be associated with COVID-19 (a disease can represent a symptom associated with COVID-19 or a disease that occurs concurrently with COVID-19). In path b, we also defined that there must be an association between the 2 drugs. The results of the 3 paths were finally ranked by the Adamic Adar score, where the results of path a contained 49 chemicals, the results of path b contained 97 chemicals, and the results of path c contained 9 chemicals. The list of results and the entire graphs of the 3 paths (JSON format) are available online [54]. As shown in Figure 4D, we also provide an example for each of the 3 paths. In path a, revefenacin was a drug for chronic obstructive pulmonary disease (COPD), which can help relax the lung muscles and help relieve cough and shortness of breath [55], while COPD has many potential negative interactions with COVID-19 [56] and abnormal expression of angiotensin converting enzyme 2 (ACE2) plays an important role in both COPD and COVID-19 [57,58]. Since revefenacin and COVID-19 did not appear together in all literature abstracts, we did a full-text search review and found that Djokovic et al [59] used structure-based molecular modeling and physiological-based pharmacokinetic modeling for drug repurposing, and the full text mentions revefenacin as a candidate with potential activity on the SARS-CoV-2 main protease. In path b, we focused the second node of the disease on a specific symptom (gastrointestinal symptom), and rabeprazole and omeprazole have been used to treat gastrointestinal diseases and have the same type of efficacy [60,61], while omeprazole has been used to treat COVID-19 [62]. Rabeprazole was also mentioned in the full text of the study by Ray et al [63] as a possible treatment for COVID-19, either alone or in combination with other drugs. In path c, SB203580 can affect p38 [64], while abnormalities in p38, IL-6, and tumor necrosis factor-α (TNFα) have all been shown to be associated with COVID-19 [65-67]. In addition, p38 has also been shown to be associated with IL-6 and TNFα activity [68]. We also found that the potential treatment of inflammation with SB203580 as a p38 inhibitor has been discussed in detail in the full text of the review on COVID-19 by Malekinejad et al [69].

We also filtered out long COVID articles and non-long COVID articles of “treatment” separately and extracted drugs by detecting therapeutic relations. As shown in Multimedia Appendix 8, the results based on RE indicated that 107 drugs were involved in the treatment of long COVID, 154 drugs were involved in the treatment of non-long COVID, and only 47 drugs appeared for both non-long COVID and long COVID. For the CoronaCentral data (downloaded on March 27, 2023) [21], we retrieved coronavirus-specific entity types, including viral lineages, risk factors, symptoms, and prevention methods, along with general entities, like drugs and diseases, from all SARS-CoV-2 articles to extract relations between them. Finally, we built a set of SARS-CoV-2–specific knowledge triples. Benefiting from these coronavirus-specific entity types, the RE was applied to extract more detailed results. The CoronaCentral data have been made available [70].

## Discussion

### Principal Findings

In this study, we comprehensively investigated biomedical RE from the perspectives of data, model, and application. The study conducted performance analyses for different entity information levels, pretrained model architectures, and domain models. We also proposed a continued pretraining model for the specific RE task, which achieved the best performance for a single model. The RE models were then integrated as a convenient Docker tool with applications to practical biomedical problems. LitCovid was used to obtain a corpus of literature sorted by topic. Relation enrichment and correlation analysis for 8 types of topics demonstrated that there were differences in relation type between article topics, indicating the biological significance of text mining from the perspective of RE. For articles on treatment and mechanism topics, the output relation triples between key entities were constructed as a relation graph database, which not only allowed us to obtain known therapeutic drugs from existing research, but also helped us to perform drug repurposing via link algorithms and predict novel drug paths. In addition, RE on the treatment corpus of non-long COVID and long COVID could help us to pinpoint the therapeutic drug differences between them. In order to apply RE more profoundly, we also extracted relations between coronavirus-specific entities, like symptoms, viral lineages, and risk factors, from CoronaCentral, giving us a more precise knowledge network of the coronavirus. 

The data set we applied consisted of 125 biomedical relations covering treatment, components, side effects, metabolic mechanisms, etc. All relation types and example sentences are presented in Multimedia Appendix 9. Compared to existing biomedical data sets, such as ChemProt [71,72], DDI [73], and GAD [74], data sets integrating BioRel and UMLS contain significantly more words, entities, and relations. The experiments on data sets with different entity levels showed that relation prediction was the most accurate when the input contained the semantic type name of the entity. Additionally, the entity types with a unique identifier (such as “T047” and “DISO,” which appear in code and abbreviation formats) performed worse than those with a full expression, and could result in the wrong prediction. Finally, although semantic type group can categorize all semantic types, the information is also more likely to lead to an incorrect prediction. Taken together, these results suggest that more detailed information on entity types with semantic information can help to significantly improve the accuracy of RE.

In model comparison, CT_PubMedBERT achieved the best performance, and biomedical domain models had better performance than general models. The reason was that the model pretrained with biomedical domain-specific literature corpora and unlabeled task-specific data sets had the most extensive background information, giving it a more precise handle on the meaning of individual words. Domain models also integrated the contextual information of sentences into the word vector owing to the use of domain-specific vocabulary and pretraining from scratch (as opposed to the Word2Vec model [75,76]). Moreover, the ensemble model improved the overall RE performance, as confirmed by the performance indicators.

In practical applications, different data sources (literature) may lead to controversial statistical analysis results. For example, hydroxychloroquine has been increasingly found to be useless in the treatment of COVID-19 [77], but many studies, especially early stage studies, include a description that it is a potential and safe drug [78-80]. Therefore, data preprocessing is critical to the quality of RE results. For example, to find more promising drug–COVID-19 treatment triples, 3 preprocessing steps were used. First, papers within the last year (June 1, 2022, to June 1, 2023) were selected to remove studies in the early stage of the pandemic that were not in-depth. Second, the Altmetric score of each paper was crawled, which is an important indicator of research attention, and papers with higher-than-average scores (about 10% of all papers) were used. Third, a rule-based approach was used to increase credibility by selecting text with conclusive descriptions in the abstract. According to these criteria, the top 3 drugs ranked by the sum of the probability scores included ritonavir, dexamethasone, and baricitinib, which are currently widely used and validated in the treatment of COVID-19 [81].

Biomedical and clinical researchers typically keep track of new discoveries through extensive collections of scientific articles, and language model–based NLP techniques are of great help to researchers in extracting information of interest [82]. Research into KG construction, graph path prediction, and automatic text summary greatly benefits from the automated RE process [83,84]. Building a KG can be very tedious and time-consuming if the entities and relations need to be manually identified and inputted. The RE models and tools we built improve the development of large-scale biomedical RE and enable the automatic extraction of relations from scientific articles. These tools make it possible to rapidly build and update a KG. More importantly, applying graph algorithms to KGs enables knowledge discovery, and the representations of KGs can be used for many downstream tasks. The large-scale RE in biomedical text mining can help inform future research, and rigorous conclusions can be drawn through further experiments.

The statistics of the error analysis involved the CT_PubMedBERT model mispredicting the gold standard relation type as other relation types. Multimedia Appendix 10 shows the top 100 misprediction types by percentage of total errors, and the mispredictions can be mainly divided into 3 categories. The first category included mispredicting the direction of the relation, such as the first (7.03%) and second (5.95%) ranked misprediction types (“anatomic structure is physical part of” and “has physical part of anatomic structure”), which both express anatomic structure part relations but in different directions. The second category included confusing relation types involving subordinate meanings with “not a relation,” such as “nichd parent of” and “chemical structure of,” indicating that the model may be less effective in distinguishing subordinate relation types from no relation. The third category included errors in distinguishing similar relation types, such as “disease has primary anatomic site” and “disease has associated anatomic site,” both of which mean that the disease has an anatomic site. Therefore, the improvement of these 3 categories of errors is a direction for future research, for example, data augmentation may improve the second and third categories, while manual review to generate custom rules may improve the first category.

### Limitations

Our study provides a biomedical RE implementation that makes analysis accessible to the research community. Nevertheless, several limitations exist. First, the rare entity type and unbalanced data distribution of relation categories limit the performance of the RE. For example, “associated with malfunction of gene product,” “biomarker type includes gene product,” and “gene product has structural domain or motif” had a relatively small amount of data but achieved F1-scores above 0.95 because semantic features in these relations are usually unambiguous and distinctive. The relations “anatomic structure is physical part of,” “disease has associated anatomic site,” and “may treat” achieved comparatively high F1-scores owing to the greater amount of data. In addition, we observed multiple errors and reversed mistakes between relations. Our RE model would benefit the most from optimizations to the data set and algorithm. Short sentences include fewer words and might not provide the RE model with detailed information for correct prediction. Domain-specific expressions, such as formula symbols, measurement units, and proper nouns, are frequently used in scientific writing. Therefore, incorporating customized rules into biomedical RE will increase the efficiency of the model. To improve performance by enhancing semantic features, effective data augmentation might also be used for relation types with small data sizes.

### Conclusions

Our study broke new ground in the pretrained language model it used, the comprehensiveness of its biomedical RE topics, the many types of relations it covered, and the insights it generated into hot and prolific scientific topics. We not only evaluated the impact of entity semantic richness, but also compared different model architectures and domain models. We also proposed a continued pretraining model for our specific RE task and fine-tuned it to achieve the best performance. The RE models were packaged as an easy-to-use tool and were applied to the COVID-19 corpus for usability analysis. Furthermore, our relation graph database pipeline can be applied to other large-scale biomedical text mining areas of interest and is not intrinsically limited to cases as shown in this study. It is our hope that our contributions to the field of knowledge mining and the presented tools will facilitate other biomedical and clinical research in the future.

## Appendix

Multimedia Appendix 1 Examples of relation types and sentences.

Multimedia Appendix 2 Formulae used in the study.

Multimedia Appendix 3 F1-score difference of each relation type for the 4 entity information levels compared to the no semantic type.

Multimedia Appendix 4 Performance of CT_PubMedBERT for each relation type.

Multimedia Appendix 5 F1-score difference of CT_PubMedBERT compared with PubMedBERT for each relation type.

Multimedia Appendix 6 F1-score and loss trend for 100 epochs.

Multimedia Appendix 7 Relation graph database visualization.

Multimedia Appendix 8 Venn diagram of drugs between non-long COVID and long COVID.

Multimedia Appendix 9 All relation types and example sentences.

Multimedia Appendix 10 Top 100 misprediction types by percentage.

## Abbreviations

COPD: chronic obstructive pulmonary disease
CUI: Concept Unique Identifier
IL-6: interleukin-6
KG: knowledge graph
MLM: masked language modeling
NLP: natural language processing
PCA: principal component analysis
RE: relation extraction
RRF: rich release format
TNFα: tumor necrosis factor-α
UMLS: Unified Medical Language System

## Data Availability

The following data have been made available: Neo4j data of the COVID-19 relation graph database [48], novel drug path data [54], and CoronaCentral data [70].
