# Supplementary material for: Large-Scale Biomedical Relation Extraction Across Diverse Relation Types: Model Development and Usability Study on COVID-19
Source: J Med Internet Res. 2023 Sep 20;25:e48115. doi: 10.2196/48115 (PMC10551783; doi:10.2196/48115)
Supplement: Multimedia Appendix 1 [file jmir_v25i1e48115_app1.docx]

**Multimedia Appendix 1.** Examples of relation types and sentences.

| Relation type | Sentence |
| --- | --- |
| not_a_relation | Prolonged fasting hypoglycemia due to **insulin antibodies** in patient with non-insulin-dependent diabetes mellitus: effect of insulin withdrawal on insulin-**antibody**-binding kinetics. |
| induced_by | The authors report five cases of **mydriasis** induced by the use of transdermal **scopolamine** delivery systems. |
| induces | Reversal of **scopolamine**-induced **amnesia** and alterations in energy metabolism by the nootropic piracetam: implications regarding identification of brain structures involved in consolidation of memory traces. |
| may_be_associated_disease_of_disease | **Malignant schwannoma** usually is associated with **neurofibromatosis** and has a poor prognosis, spreading along the nerve of origin or by blood stream. |
| may_be_diagnosed_by | In 37 patients with suspected **vasospastic angina** diagnostic **ergonovine** testing was used as a method of analysing the efficacy of sublingual isdn in comparison with sublingual and intracoronary nifedipine on coronary luminal diameter, left ventricular ejection fraction, aortic blood pressure, heart rate and left ventricular end-diastolic pressure. |
| may_be_finding_of_disease | The classic triad of hematuria, **flank pain** and abdominal mass is present in only 15 percent of patients with **adenocarcinoma of the kidney**. |
| may_be_prevented_by | To prevent **ventricular tachycardia** or when treatment is indicated for ventricular premature beats, ajmaline, **propafenon**, quinidine, disopyramid or mexiletine, occasionally in combination with a beta-adrenergic blocker should be employed. |
| may_be_treated_by | **Duodenal ulcer** caused by increased acid production (pentagastrin plus carbachol induced) was suppressed by pirenzepine, doxepin and **cimetidine**. |
| may_diagnose | Evaluation of in vitro lymphoblastoid transformation in the presence of **purified protein derivative** as a diagnostic test in **tuberculosis**. |
| may_prevent | Large-scale purification of **hepatitis b surface antigen**, applicable to the preparation of potential vaccines for prevention of **hepatitis b**, is described. |
| may_treat | **Norfloxacin** is a valuable addition to the oral antimicrobial agents available for the treatment of **urinary tract infection**. |
