# Supplementary material for: Large-Scale Biomedical Relation Extraction Across Diverse Relation Types: Model Development and Usability Study on COVID-19
Source: J Med Internet Res. 2023 Sep 20;25:e48115. doi: 10.2196/48115 (PMC10551783; doi:10.2196/48115)
Supplement: Multimedia Appendix 2 [file jmir_v25i1e48115_app2.docx]

**Multimedia** **Appendix 2.** Formulae used in the study.

The formulae of evaluation metrics were presented below, for 125 relation types: $R_{1}$*, ……,* $R_{i}$*, ……,* $R_{125}$,

the precision of relation type $i$ is defined as:

$$\begin{aligned} {Precision}^{i}=\frac{{The number of samples correctly predicted as R}_{i}}{the number of samples predicted as R_{i}}\#\left( 1 \right) \end{aligned}$$

the recall of relation type $i$ is defined as:

$$\begin{aligned} {Recall}^{i}=\frac{{The number of samples correctly predicted as R}_{i}}{the number of samples of R_{i}}\#\left( 2 \right) \end{aligned}$$

the F1 score of relation type $i$ is defined as:

$$\begin{aligned} {F1-score}^{i}=\frac{2*{Precision}^{i}*{Recall}^{i}}{{Precision}^{i}+{Recall}^{i}}\#\left( 3 \right) \end{aligned}$$

The calculation of Adamic Adar scores is presented below:

$$\begin{aligned} Adamic Adar scores\left( chemical,disease \right)= \sum_{u\in N\left( chemical \right)\cap N\left( disease \right)} \frac{1}{\log\left| N\left( u \right) \right|}\#\left( 4 \right) \end{aligned}$$

where $N(u)$ is the set of nodes adjacent to $u$.
