# Supplementary material for: Large-Scale Biomedical Relation Extraction Across Diverse Relation Types: Model Development and Usability Study on COVID-19
Source: J Med Internet Res. 2023 Sep 20;25:e48115. doi: 10.2196/48115 (PMC10551783; doi:10.2196/48115)
Supplement: Multimedia Appendix 3 [file jmir_v25i1e48115_app3.docx]

**Multimedia Appendix 3.** F1-score difference of each relation type for the 4 entity information levels compared to the no semantic type.

| Relation type | Semantic type group abbreviation | Semantic type group | Semantic type code | Semantic type |
| --- | --- | --- | --- | --- |
| not a relation | 0.0498 | 0.0531 | 0.0859 | 0.0997 |
| active_metabolites_of | 0.1718 | 0.1789 | -0.1579 | 0.3967 |
| anatomic_structure_has_location | 0.0265 | 0.0527 | 0.3109 | 0.3706 |
| anatomic_structure_is_physical_part_of | 0.0535 | 0.0584 | 0.1267 | 0.1373 |
| anatomy_originated_from_biological_process | 0.1901 | 0.1791 | 0.3574 | 0.3792 |
| associated_with_malfunction_of_gene_product | 0.2364 | 0.2577 | 0.7240 | 0.8500 |
| biological_process_has_associated_location | 0.1317 | 0.1344 | 0.1414 | 0.1450 |
| biological_process_has_initiator_chemical_or_drug | 0.0797 | 0.0891 | 0.2126 | 0.2180 |
| biological_process_has_initiator_process | 0.0000 | 0.2556 | 0.6358 | 0.6478 |
| biological_process_has_result_anatomy | 0.1792 | 0.1955 | 0.3548 | 0.4001 |
| biological_process_has_result_biological_process | 0.4923 | 0.4923 | 0.5235 | 0.4883 |
| biological_process_has_result_chemical_or_drug | 0.0853 | 0.0845 | 0.2119 | 0.2179 |
| biological_process_involves_gene_product | 0.1651 | 0.1688 | 0.2307 | 0.2318 |
| biological_process_is_part_of_process | 0.1878 | 0.1959 | 0.3581 | 0.3823 |
| biological_process_results_from_biological_process | 0.2647 | 0.2775 | 0.3838 | 0.4178 |
| biomarker_type_includes_gene_product | 0.0887 | 0.0655 | 0.2397 | 0.2593 |
| cdrh_parent_of | 0.1560 | 0.1266 | 0.2989 | 0.3369 |
| chemical_or_drug_affects_gene_product | 0.0536 | 0.1111 | 0.6349 | 0.7111 |
| chemical_or_drug_initiates_biological_process | 0.1366 | 0.1275 | 0.2280 | 0.2299 |
| chemical_or_drug_is_product_of_biological_process | 0.1067 | 0.0969 | 0.2496 | 0.2483 |
| chemical_structure_of | -0.0254 | -0.0153 | 0.1538 | 0.1802 |
| chemotherapy_regimen_has_component | 0.0862 | 0.0862 | 0.0862 | 0.0862 |
| completely_excised_anatomy_has_procedure | 0.0000 | 0.0000 | 0.0000 | 0.4667 |
| complex_has_physical_part | 0.2181 | 0.1556 | 0.2482 | 0.4668 |
| concept_in_subset | 0.3777 | 0.4224 | 0.5005 | 0.4915 |
| conceptual_part_of | 0.0054 | 0.0163 | 0.0126 | 0.0236 |
| contraindicated_with_disease | 0.0754 | 0.0705 | 0.1137 | 0.1665 |
| contraindicating_class_of | 0.0466 | 0.0170 | 0.1979 | 0.2219 |
| disease_excludes_normal_cell_origin | 0.0000 | 0.0000 | 0.0000 | 0.0870 |
| disease_excludes_primary_anatomic_site | 0.0357 | 0.2205 | 0.0121 | 0.2381 |
| disease_has_abnormal_cell | 0.1742 | 0.2030 | 0.2243 | 0.2805 |
| disease_has_associated_anatomic_site | 0.1222 | 0.1233 | 0.1478 | 0.1628 |
| disease_has_associated_disease | 0.0000 | 0.0000 | 0.0526 | 0.2182 |
| disease_has_associated_gene | 0.4958 | 0.5878 | 0.5636 | 0.6196 |
| disease_has_finding | 0.0526 | 0.0867 | 0.4824 | 0.5145 |
| disease_has_metastatic_anatomic_site | 0.1838 | 0.3044 | 0.3399 | 0.5989 |
| disease_has_normal_cell_origin | 0.1206 | 0.1545 | 0.2436 | 0.2637 |
| disease_has_normal_tissue_origin | 0.0557 | 0.1466 | 0.4787 | 0.4874 |
| disease_has_primary_anatomic_site | 0.0810 | 0.0842 | 0.1603 | 0.1846 |
| disease_may_have_associated_disease | 0.0428 | 0.0227 | 0.1579 | 0.1855 |
| disease_may_have_finding | -0.2217 | 0.0496 | 0.2647 | 0.3669 |
| excised_anatomy_has_procedure | 0.0842 | 0.0850 | 0.1348 | 0.1628 |
| gene_associated_with_disease | 0.4996 | 0.5120 | 0.5686 | 0.5747 |
| gene_encodes_gene_product | 0.5067 | 0.5158 | 0.5279 | 0.5279 |
| gene_found_in_organism | 0.2306 | 0.2305 | 0.2306 | 0.2321 |
| gene_mapped_to_disease | 0.0000 | 0.0000 | 0.3810 | 0.5000 |
| gene_plays_role_in_process | 0.4000 | 0.4050 | 0.4075 | 0.4049 |
| gene_product_affected_by_chemical_or_drug | 0.2500 | 0.3636 | 0.9167 | 0.9796 |
| gene_product_encoded_by_gene | 0.6254 | 0.6222 | 0.6592 | 0.6522 |
| gene_product_expressed_in_tissue | 0.1567 | 0.1570 | 0.2169 | 0.2265 |
| gene_product_has_associated_anatomy | 0.1951 | 0.1855 | 0.2566 | 0.2585 |
| gene_product_has_biochemical_function | 0.0171 | 0.0366 | 0.1651 | 0.2284 |
| gene_product_has_chemical_classification | 0.0383 | 0.0103 | 0.2398 | 0.3079 |
| gene_product_has_organism_source | 0.1984 | 0.1958 | 0.2070 | 0.2070 |
| gene_product_has_structural_domain_or_motif | 0.4447 | 0.4229 | 0.6556 | 0.6556 |
| gene_product_is_biomarker_of | 0.1538 | 0.1231 | 0.3019 | 0.3744 |
| gene_product_is_physical_part_of | 0.2868 | 0.1168 | 0.1618 | 0.4281 |
| gene_product_malfunction_associated_with_disease | 0.2047 | 0.1211 | 0.3813 | 0.4283 |
| gene_product_plays_role_in_biological_process | 0.1908 | 0.1852 | 0.2504 | 0.2511 |
| has_active_metabolites | -0.0163 | 0.0144 | -0.0599 | 0.0834 |
| has_cdrh_parent | 0.1012 | 0.1143 | 0.2231 | 0.2934 |
| has_chemical_structure | -0.0160 | -0.0098 | 0.2005 | 0.2463 |
| has_conceptual_part | 0.0168 | 0.0402 | 0.0288 | 0.0555 |
| has_contraindicated_drug | 0.0505 | 0.0410 | 0.1078 | 0.1650 |
| has_contraindicating_class | -0.0829 | 0.0498 | 0.2756 | 0.3215 |
| has_free_acid_or_base_form | 0.0000 | 0.0000 | 0.0000 | 0.2378 |
| has_ingredient | -0.0556 | -0.0338 | 0.4220 | 0.4668 |
| has_mechanism_of_action | 0.5226 | 0.6334 | 0.6702 | 0.6877 |
| has_nichd_parent | 0.0683 | 0.0739 | 0.1130 | 0.1390 |
| has_physical_part_of_anatomic_structure | 0.0561 | 0.0595 | 0.1239 | 0.1332 |
| has_physiologic_effect | 0.2145 | 0.2510 | 0.3480 | 0.3641 |
| has_salt_form | 0.0000 | 0.0580 | 0.0000 | 0.1667 |
| has_therapeutic_class | 0.0137 | 0.0308 | 0.1679 | 0.1781 |
| has_tradename | 0.0018 | 0.0026 | 0.0394 | 0.0742 |
| induced_by | 0.1357 | 0.0605 | 0.4100 | 0.5470 |
| induces | 0.0476 | 0.0513 | 0.1429 | 0.7353 |
| ingredient_of | 0.0141 | 0.0860 | 0.5738 | 0.6153 |
| is_abnormal_cell_of_disease | 0.1042 | 0.1475 | 0.1450 | 0.2395 |
| is_associated_anatomic_site_of | 0.1130 | 0.1149 | 0.1381 | 0.1515 |
| is_associated_anatomy_of_gene_product | 0.1902 | 0.1901 | 0.2680 | 0.2752 |
| is_associated_disease_of | 0.0000 | 0.0000 | 0.0000 | 0.2917 |
| is_biochemical_function_of_gene_product | 0.0414 | 0.0464 | 0.1531 | 0.2141 |
| is_chemical_classification_of_gene_product | 0.0276 | 0.0592 | 0.2865 | 0.3219 |
| is_component_of_chemotherapy_regimen | 0.0772 | 0.0772 | 0.0772 | 0.0772 |
| is_finding_of_disease | 0.0140 | 0.0299 | 0.2751 | 0.3165 |
| is_location_of_anatomic_structure | 0.0524 | 0.0992 | 0.2436 | 0.3067 |
| is_location_of_biological_process | 0.1413 | 0.1414 | 0.1505 | 0.1504 |
| is_marked_by_gene_product | 0.2668 | 0.2270 | 0.2218 | 0.3737 |
| is_metastatic_anatomic_site_of_disease | 0.1311 | 0.3143 | 0.3733 | 0.5625 |
| is_normal_cell_origin_of_disease | 0.0899 | 0.1141 | 0.1880 | 0.2229 |
| is_normal_tissue_origin_of_disease | -0.0065 | 0.0553 | 0.3959 | 0.4100 |
| is_not_normal_cell_origin_of_disease | 0.0000 | 0.0000 | 0.0000 | 0.1143 |
| is_not_primary_anatomic_site_of_disease | 0.3710 | 0.3021 | 0.4157 | 0.4785 |
| is_organism_source_of_gene_product | 0.2232 | 0.2253 | 0.2295 | 0.2281 |
| is_physiologic_effect_of_chemical_or_drug | 0.0000 | 0.3238 | 0.4137 | 0.5366 |
| is_primary_anatomic_site_of_disease | 0.0631 | 0.0649 | 0.1415 | 0.1734 |
| is_structural_domain_or_motif_of_gene_product | 0.2797 | 0.3034 | 0.4264 | 0.4277 |
| may_be_associated_disease_of_disease | 0.0219 | 0.1209 | 0.1392 | 0.2735 |
| may_be_diagnosed_by | 0.1800 | 0.2102 | 0.2561 | 0.3182 |
| may_be_finding_of_disease | 0.0000 | 0.0000 | 0.3714 | 0.6154 |
| may_be_prevented_by | 0.0784 | 0.0710 | 0.1626 | 0.1962 |
| may_be_treated_by | 0.0391 | 0.0404 | 0.0538 | 0.0630 |
| may_diagnose | 0.1739 | 0.1359 | 0.3136 | 0.3638 |
| may_prevent | 0.0616 | 0.0731 | 0.1720 | 0.2152 |
| may_treat | 0.0340 | 0.0319 | 0.0507 | 0.0618 |
| mechanism_of_action_of | 0.1513 | 0.1983 | 0.2495 | 0.2495 |
| nichd_parent_of | 0.0724 | 0.0809 | 0.1267 | 0.1570 |
| organism_has_gene | 0.2886 | 0.2900 | 0.2900 | 0.2872 |
| partially_excised_anatomy_has_procedure | 0.1097 | 0.1461 | 0.1582 | 0.1954 |
| pathogenesis_of_disease_involves_gene | 0.0861 | 0.0379 | 0.1863 | 0.2670 |
| physiologic_effect_of | 0.2492 | 0.2598 | 0.3836 | 0.4052 |
| procedure_has_completely_excised_anatomy | 0.0000 | 0.0000 | 0.0000 | 0.5143 |
| procedure_has_excised_anatomy | 0.0618 | 0.0858 | 0.1090 | 0.1415 |
| procedure_has_partially_excised_anatomy | 0.1614 | 0.2424 | 0.2791 | 0.3581 |
| procedure_has_target_anatomy | 0.1351 | 0.1251 | 0.1797 | 0.2407 |
| process_includes_biological_process | 0.1965 | 0.1971 | 0.3415 | 0.3586 |
| process_initiates_biological_process | 0.0421 | 0.0943 | 0.2707 | 0.2897 |
| process_involves_gene | 0.4649 | 0.4649 | 0.4677 | 0.4647 |
| product_component_of | 0.0000 | 0.0000 | 0.4444 | 0.6087 |
| special_category_includes_neoplasm | 0.2667 | 0.2581 | 0.1379 | 0.4390 |
| subset_includes_concept | 0.3040 | 0.3418 | 0.4105 | 0.4039 |
| target_anatomy_has_procedure | 0.1861 | 0.2043 | 0.2873 | 0.3508 |
| therapeutic_class_of | 0.0236 | 0.0227 | 0.1270 | 0.1301 |
| tissue_is_expression_site_of_gene_product | 0.1910 | 0.1927 | 0.2540 | 0.2610 |
| tradename_of | -0.0571 | 0.0730 | -0.0742 | 0.2227 |
