# Supplementary material for: Large-Scale Biomedical Relation Extraction Across Diverse Relation Types: Model Development and Usability Study on COVID-19
Source: J Med Internet Res. 2023 Sep 20;25:e48115. doi: 10.2196/48115 (PMC10551783; doi:10.2196/48115)
Supplement: Multimedia Appendix 4 [file jmir_v25i1e48115_app4.docx]

**Multimedia Appendix 4**. Performance of CT_PubMedBERT for each relation type.

| Relation type | Precision | Recall | F1-score | Support |
| --- | --- | --- | --- | --- |
| not_a_relation | 0.9204 | 0.9238 | 0.9221 | 27801 |
| active_metabolites_of | 0.6226 | 0.5000 | 0.5546 | 66 |
| anatomic_structure_has_location | 0.6702 | 0.6063 | 0.6367 | 315 |
| anatomic_structure_is_physical_part_of | 0.9301 | 0.9210 | 0.9255 | 11398 |
| anatomy_originated_from_biological_process | 0.7770 | 0.7718 | 0.7744 | 149 |
| associated_with_malfunction_of_gene_product | 0.9565 | 0.8800 | 0.9167 | 25 |
| biological_process_has_associated_location | 0.9919 | 0.9870 | 0.9894 | 4071 |
| biological_process_has_initiator_chemical_or_drug | 0.9958 | 0.9813 | 0.9885 | 482 |
| biological_process_has_initiator_process | 0.7234 | 0.7391 | 0.7312 | 46 |
| biological_process_has_result_anatomy | 0.7650 | 0.8235 | 0.7932 | 170 |
| biological_process_has_result_biological_process | 0.8261 | 0.6552 | 0.7308 | 29 |
| biological_process_has_result_chemical_or_drug | 0.9728 | 0.9949 | 0.9837 | 395 |
| biological_process_involves_gene_product | 0.9962 | 0.9947 | 0.9954 | 1319 |
| biological_process_is_part_of_process | 0.8613 | 0.8923 | 0.8765 | 362 |
| biological_process_results_from_biological_process | 0.8000 | 0.5517 | 0.6531 | 29 |
| biomarker_type_includes_gene_product | 1.0000 | 0.9286 | 0.9630 | 28 |
| cdrh_parent_of | 0.5111 | 0.4510 | 0.4792 | 204 |
| chemical_or_drug_affects_gene_product | 1.0000 | 0.8750 | 0.9333 | 24 |
| chemical_or_drug_initiates_biological_process | 0.9945 | 0.9927 | 0.9936 | 548 |
| chemical_or_drug_is_product_of_biological_process | 0.9875 | 0.9900 | 0.9887 | 399 |
| chemical_structure_of | 0.8049 | 0.8337 | 0.8190 | 1341 |
| chemotherapy_regimen_has_component | 1.0000 | 1.0000 | 1.0000 | 233 |
| completely_excised_anatomy_has_procedure | 0.6364 | 0.3684 | 0.4667 | 19 |
| complex_has_physical_part | 0.5800 | 0.6744 | 0.6237 | 43 |
| concept_in_subset | 1.0000 | 0.9483 | 0.9735 | 58 |
| conceptual_part_of | 0.9469 | 0.9382 | 0.9425 | 437 |
| contraindicated_with_disease | 0.8791 | 0.7889 | 0.8315 | 1023 |
| contraindicating_class_of | 0.6867 | 0.5278 | 0.5969 | 108 |
| disease_excludes_normal_cell_origin | 0.2857 | 0.0513 | 0.0870 | 39 |
| disease_excludes_primary_anatomic_site | 0.5135 | 0.4043 | 0.4524 | 47 |
| disease_has_abnormal_cell | 0.7429 | 0.8125 | 0.7761 | 288 |
| disease_has_associated_anatomic_site | 0.9021 | 0.9089 | 0.9055 | 2656 |
| disease_has_associated_disease | 0.3333 | 0.1622 | 0.2182 | 37 |
| disease_has_associated_gene | 0.8571 | 0.8824 | 0.8696 | 34 |
| disease_has_finding | 0.6627 | 0.6044 | 0.6322 | 91 |
| disease_has_metastatic_anatomic_site | 0.7073 | 0.5800 | 0.6374 | 50 |
| disease_has_normal_cell_origin | 0.8579 | 0.8638 | 0.8608 | 580 |
| disease_has_normal_tissue_origin | 0.8528 | 0.8660 | 0.8593 | 194 |
| disease_has_primary_anatomic_site | 0.8054 | 0.8082 | 0.8068 | 1434 |
| disease_may_have_associated_disease | 0.4315 | 0.5478 | 0.4828 | 115 |
| disease_may_have_finding | 0.6977 | 0.5660 | 0.6250 | 53 |
| excised_anatomy_has_procedure | 0.7789 | 0.8733 | 0.8234 | 363 |
| gene_associated_with_disease | 0.7708 | 0.9487 | 0.8506 | 39 |
| gene_encodes_gene_product | 0.9938 | 0.9938 | 0.9938 | 162 |
| gene_found_in_organism | 0.9838 | 0.9902 | 0.9870 | 306 |
| gene_mapped_to_disease | 0.7500 | 0.3750 | 0.5000 | 16 |
| gene_plays_role_in_process | 0.9948 | 0.9847 | 0.9897 | 196 |
| gene_product_affected_by_chemical_or_drug | 1.0000 | 0.9600 | 0.9796 | 25 |
| gene_product_encoded_by_gene | 0.9862 | 1.0000 | 0.9931 | 143 |
| gene_product_expressed_in_tissue | 0.9711 | 0.9711 | 0.9711 | 795 |
| gene_product_has_associated_anatomy | 0.9588 | 0.9661 | 0.9624 | 1180 |
| gene_product_has_biochemical_function | 0.7531 | 0.7101 | 0.7310 | 683 |
| gene_product_has_chemical_classification | 0.7015 | 0.7015 | 0.7015 | 134 |
| gene_product_has_organism_source | 1.0000 | 0.9843 | 0.9921 | 575 |
| gene_product_has_structural_domain_or_motif | 1.0000 | 0.9524 | 0.9756 | 21 |
| gene_product_is_biomarker_of | 0.9722 | 0.8333 | 0.8974 | 42 |
| gene_product_is_physical_part_of | 0.8235 | 0.3333 | 0.4746 | 42 |
| gene_product_malfunction_associated_with_disease | 0.8800 | 0.9362 | 0.9072 | 47 |
| gene_product_plays_role_in_biological_process | 0.9947 | 0.9970 | 0.9958 | 1322 |
| has_active_metabolites | 0.5891 | 0.7170 | 0.6468 | 106 |
| has_cdrh_parent | 0.5644 | 0.4000 | 0.4682 | 230 |
| has_chemical_structure | 0.7672 | 0.7618 | 0.7645 | 424 |
| has_conceptual_part | 0.8693 | 0.8872 | 0.8782 | 195 |
| has_contraindicated_drug | 0.8247 | 0.7671 | 0.7949 | 730 |
| has_contraindicating_class | 0.5625 | 0.5567 | 0.5596 | 97 |
| has_free_acid_or_base_form | 0.3400 | 0.1828 | 0.2378 | 93 |
| has_ingredient | 0.7542 | 0.8050 | 0.7787 | 282 |
| has_mechanism_of_action | 0.9355 | 0.9667 | 0.9508 | 30 |
| has_nichd_parent | 0.7861 | 0.8138 | 0.7997 | 4490 |
| has_physical_part_of_anatomic_structure | 0.9275 | 0.9381 | 0.9328 | 12584 |
| has_physiologic_effect | 1.0000 | 0.9545 | 0.9767 | 66 |
| has_salt_form | 0.3043 | 0.1148 | 0.1667 | 61 |
| has_therapeutic_class | 0.9448 | 0.9848 | 0.9644 | 330 |
| has_tradename | 0.7892 | 0.9031 | 0.8423 | 485 |
| induced_by | 0.7188 | 0.6765 | 0.6970 | 34 |
| induces | 0.7813 | 0.6944 | 0.7353 | 36 |
| ingredient_of | 0.8815 | 0.8207 | 0.8500 | 145 |
| is_abnormal_cell_of_disease | 0.7980 | 0.8203 | 0.8090 | 395 |
| is_associated_anatomic_site_of | 0.9082 | 0.9040 | 0.9061 | 3030 |
| is_associated_anatomy_of_gene_product | 0.9625 | 0.9617 | 0.9621 | 1122 |
| is_associated_disease_of | 0.5833 | 0.1944 | 0.2917 | 36 |
| is_biochemical_function_of_gene_product | 0.6903 | 0.7038 | 0.6970 | 719 |
| is_chemical_classification_of_gene_product | 0.6486 | 0.6486 | 0.6486 | 111 |
| is_component_of_chemotherapy_regimen | 1.0000 | 1.0000 | 1.0000 | 333 |
| is_finding_of_disease | 0.8235 | 0.7778 | 0.8000 | 108 |
| is_location_of_anatomic_structure | 0.6556 | 0.6641 | 0.6598 | 387 |
| is_location_of_biological_process | 0.9886 | 0.9884 | 0.9885 | 4138 |
| is_marked_by_gene_product | 0.9167 | 0.9565 | 0.9362 | 23 |
| is_metastatic_anatomic_site_of_disease | 0.6000 | 0.5294 | 0.5625 | 51 |
| is_normal_cell_origin_of_disease | 0.8457 | 0.8746 | 0.8599 | 614 |
| is_normal_tissue_origin_of_disease | 0.9218 | 0.9016 | 0.9116 | 183 |
| is_not_normal_cell_origin_of_disease | 1.0000 | 0.0606 | 0.1143 | 33 |
| is_not_primary_anatomic_site_of_disease | 0.6000 | 0.4565 | 0.5185 | 46 |
| is_organism_source_of_gene_product | 0.9958 | 0.9958 | 0.9958 | 714 |
| is_physiologic_effect_of_chemical_or_drug | 1.0000 | 0.9444 | 0.9714 | 18 |
| is_primary_anatomic_site_of_disease | 0.8056 | 0.8225 | 0.8140 | 1572 |
| is_structural_domain_or_motif_of_gene_product | 0.9643 | 0.9643 | 0.9643 | 28 |
| may_be_associated_disease_of_disease | 0.4651 | 0.4082 | 0.4348 | 98 |
| may_be_diagnosed_by | 0.8462 | 0.7920 | 0.8182 | 125 |
| may_be_finding_of_disease | 0.7273 | 0.5333 | 0.6154 | 45 |
| may_be_prevented_by | 0.7814 | 0.7289 | 0.7542 | 461 |
| may_be_treated_by | 0.9248 | 0.9476 | 0.9361 | 3246 |
| may_diagnose | 0.8889 | 0.8696 | 0.8791 | 138 |
| may_prevent | 0.7625 | 0.7180 | 0.7396 | 617 |
| may_treat | 0.9315 | 0.9627 | 0.9468 | 4579 |
| mechanism_of_action_of | 0.9884 | 0.9884 | 0.9884 | 86 |
| nichd_parent_of | 0.7703 | 0.7610 | 0.7656 | 4134 |
| organism_has_gene | 0.9918 | 0.9918 | 0.9918 | 366 |
| partially_excised_anatomy_has_procedure | 0.7333 | 0.6994 | 0.7160 | 173 |
| pathogenesis_of_disease_involves_gene | 0.8125 | 0.8125 | 0.8125 | 16 |
| physiologic_effect_of | 1.0000 | 0.9583 | 0.9787 | 72 |
| procedure_has_completely_excised_anatomy | 0.6923 | 0.4091 | 0.5143 | 22 |
| procedure_has_excised_anatomy | 0.7969 | 0.8844 | 0.8384 | 346 |
| procedure_has_partially_excised_anatomy | 0.8182 | 0.7452 | 0.7800 | 157 |
| procedure_has_target_anatomy | 0.8639 | 0.8194 | 0.8411 | 310 |
| process_includes_biological_process | 0.8844 | 0.8778 | 0.8811 | 401 |
| process_initiates_biological_process | 0.6667 | 0.6296 | 0.6476 | 54 |
| process_involves_gene | 0.9940 | 0.9821 | 0.9880 | 168 |
| product_component_of | 0.7778 | 0.5000 | 0.6087 | 14 |
| special_category_includes_neoplasm | 0.6000 | 0.3462 | 0.4390 | 26 |
| subset_includes_concept | 0.9865 | 0.9733 | 0.9799 | 75 |
| target_anatomy_has_procedure | 0.8830 | 0.7986 | 0.8387 | 293 |
| therapeutic_class_of | 0.9698 | 0.9948 | 0.9822 | 776 |
| tissue_is_expression_site_of_gene_product | 0.9659 | 0.9735 | 0.9697 | 756 |
| tradename_of | 0.6667 | 0.3960 | 0.4969 | 101 |
| accuracy |  |  | 0.9010 | 114565 |
| macro avg | 0.8163 | 0.7658 | 0.7813 | 114565 |
| weighted avg | 0.8998 | 0.9010 | 0.8998 | 114565 |
