# Supplementary material for: Large-Scale Biomedical Relation Extraction Across Diverse Relation Types: Model Development and Usability Study on COVID-19
Source: J Med Internet Res. 2023 Sep 20;25:e48115. doi: 10.2196/48115 (PMC10551783; doi:10.2196/48115)
Supplement: Multimedia Appendix 5 [file jmir_v25i1e48115_app5.docx]

**Multimedia Appendix 5.** F1-score difference of CT_PubMedBERT compared with PubMedBERT for each relation type.

| Relation type | F1-score difference | % of f1-score change | % of relation type in dataset | F1-score <0.5 |
| --- | --- | --- | --- | --- |
| is_not_normal_cell_origin_of_disease | 0.1143 | - | 0.0288 | 1 |
| is_associated_disease_of | 0.1250 | 75.0000 | 0.0314 | 1 |
| has_salt_form | 0.0641 | 62.5000 | 0.0532 | 1 |
| is_metastatic_anatomic_site_of_disease | 0.0919 | 19.5313 | 0.0445 | 1 |
| induces | 0.1176 | 19.0476 | 0.0314 | 0 |
| disease_excludes_primary_anatomic_site | 0.0573 | 14.5089 | 0.0410 | 1 |
| disease_has_associated_disease | 0.0259 | 13.4545 | 0.0323 | 1 |
| associated_with_malfunction_of_gene_product | 0.1082 | 13.3772 | 0.0218 | 0 |
| has_free_acid_or_base_form | 0.0272 | 12.9371 | 0.0812 | 1 |
| disease_has_metastatic_anatomic_site | 0.0689 | 12.1286 | 0.0436 | 0 |
| active_metabolites_of | 0.0546 | 10.9244 | 0.0576 | 0 |
| complex_has_physical_part | 0.0590 | 10.4391 | 0.0375 | 0 |
| has_cdrh_parent | 0.0425 | 9.9710 | 0.2008 | 1 |
| tradename_of | 0.0441 | 9.7308 | 0.0882 | 1 |
| disease_may_have_finding | 0.0505 | 8.7963 | 0.0463 | 0 |
| has_chemical_structure | 0.0603 | 8.5638 | 0.3701 | 0 |
| cdrh_parent_of | 0.0376 | 8.5172 | 0.1781 | 1 |
| chemical_or_drug_affects_gene_product | 0.0697 | 8.0702 | 0.0209 | 0 |
| has_active_metabolites | 0.0468 | 7.8014 | 0.0925 | 0 |
| may_be_finding_of_disease | 0.0440 | 7.6923 | 0.0393 | 0 |
| anatomy_originated_from_biological_process | 0.0539 | 7.4766 | 0.1301 | 0 |
| anatomic_structure_has_location | 0.0306 | 5.0500 | 0.2750 | 0 |
| gene_product_has_chemical_classification | 0.0325 | 4.8507 | 0.1170 | 0 |
| procedure_has_partially_excised_anatomy | 0.0334 | 4.4771 | 0.1370 | 0 |
| is_marked_by_gene_product | 0.0382 | 4.2553 | 0.0201 | 0 |
| has_contraindicated_drug | 0.0274 | 3.5699 | 0.6372 | 0 |
| is_finding_of_disease | 0.0273 | 3.5294 | 0.0943 | 0 |
| gene_product_has_biochemical_function | 0.0246 | 3.4877 | 0.5962 | 0 |
| may_be_diagnosed_by | 0.0265 | 3.3493 | 0.1091 | 0 |
| procedure_has_target_anatomy | 0.0272 | 3.3448 | 0.2706 | 0 |
| may_be_prevented_by | 0.0244 | 3.3411 | 0.4024 | 0 |
| has_mechanism_of_action | 0.0302 | 3.2787 | 0.0262 | 0 |
| is_biochemical_function_of_gene_product | 0.0215 | 3.1774 | 0.6276 | 0 |
| may_prevent | 0.0218 | 3.0416 | 0.5386 | 0 |
| is_location_of_anatomic_structure | 0.0187 | 2.9207 | 0.3378 | 0 |
| pathogenesis_of_disease_involves_gene | 0.0230 | 2.9167 | 0.0140 | 0 |
| is_physiologic_effect_of_chemical_or_drug | 0.0270 | 2.8571 | 0.0157 | 0 |
| gene_product_has_structural_domain_or_motif | 0.0256 | 2.6958 | 0.0183 | 0 |
| is_abnormal_cell_of_disease | 0.0204 | 2.5872 | 0.3448 | 0 |
| ingredient_of | 0.0202 | 2.4359 | 0.1266 | 0 |
| has_conceptual_part | 0.0203 | 2.3669 | 0.1702 | 0 |
| biological_process_results_from_biological_process | 0.0148 | 2.3129 | 0.0253 | 0 |
| chemical_structure_of | 0.0185 | 2.3053 | 1.1705 | 0 |
| physiologic_effect_of | 0.0216 | 2.2547 | 0.0628 | 0 |
| has_tradename | 0.0184 | 2.2380 | 0.4233 | 0 |
| contraindicated_with_disease | 0.0170 | 2.0847 | 0.8929 | 0 |
| disease_has_associated_gene | 0.0171 | 2.0067 | 0.0297 | 0 |
| has_ingredient | 0.0143 | 1.8727 | 0.2461 | 0 |
| biological_process_has_result_anatomy | 0.0142 | 1.8223 | 0.1484 | 0 |
| nichd_parent_of | 0.0135 | 1.7897 | 3.6084 | 0 |
| disease_may_have_associated_disease | 0.0081 | 1.6959 | 0.1004 | 1 |
| is_primary_anatomic_site_of_disease | 0.0127 | 1.5839 | 1.3721 | 0 |
| procedure_has_excised_anatomy | 0.0127 | 1.5435 | 0.3020 | 0 |
| may_be_associated_disease_of_disease | 0.0062 | 1.4493 | 0.0855 | 1 |
| is_normal_tissue_origin_of_disease | 0.0130 | 1.4435 | 0.1597 | 0 |
| target_anatomy_has_procedure | 0.0112 | 1.3590 | 0.2558 | 0 |
| biological_process_has_initiator_process | 0.0095 | 1.3210 | 0.0402 | 0 |
| has_nichd_parent | 0.0096 | 1.2189 | 3.9192 | 0 |
| may_diagnose | 0.0086 | 0.9899 | 0.1205 | 0 |
| not_a_relation | 0.0080 | 0.8734 | 24.2666 | 0 |
| gene_product_is_physical_part_of | 0.0040 | 0.8475 | 0.0367 | 1 |
| has_physiologic_effect | 0.0080 | 0.8252 | 0.0576 | 0 |
| conceptual_part_of | 0.0069 | 0.7371 | 0.3814 | 0 |
| partially_excised_anatomy_has_procedure | 0.0051 | 0.7221 | 0.1510 | 0 |
| may_treat | 0.0065 | 0.6868 | 3.9969 | 0 |
| has_physical_part_of_anatomic_structure | 0.0060 | 0.6477 | 10.9842 | 0 |
| disease_has_primary_anatomic_site | 0.0052 | 0.6434 | 1.2517 | 0 |
| disease_has_associated_anatomic_site | 0.0052 | 0.5790 | 2.3183 | 0 |
| anatomic_structure_is_physical_part_of | 0.0049 | 0.5336 | 9.9489 | 0 |
| may_be_treated_by | 0.0043 | 0.4650 | 2.8333 | 0 |
| therapeutic_class_of | 0.0033 | 0.3373 | 0.6773 | 0 |
| biological_process_involves_gene_product | 0.0030 | 0.3064 | 1.1513 | 0 |
| is_associated_anatomy_of_gene_product | 0.0026 | 0.2713 | 0.9794 | 0 |
| biological_process_has_result_chemical_or_drug | 0.0025 | 0.2551 | 0.3448 | 0 |
| chemical_or_drug_is_product_of_biological_process | 0.0025 | 0.2503 | 0.3483 | 0 |
| is_associated_anatomic_site_of | 0.0022 | 0.2379 | 2.6448 | 0 |
| biological_process_has_initiator_chemical_or_drug | 0.0022 | 0.2206 | 0.4207 | 0 |
| gene_product_malfunction_associated_with_disease | 0.0020 | 0.2158 | 0.0410 | 0 |
| chemotherapy_regimen_has_component | 0.0021 | 0.2146 | 0.2034 | 0 |
| biological_process_has_associated_location | 0.0019 | 0.1875 | 3.5534 | 0 |
| gene_product_expressed_in_tissue | 0.0018 | 0.1847 | 0.6939 | 0 |
| gene_found_in_organism | 0.0016 | 0.1629 | 0.2671 | 0 |
| is_component_of_chemotherapy_regimen | 0.0015 | 0.1502 | 0.2907 | 0 |
| is_normal_cell_origin_of_disease | 0.0012 | 0.1379 | 0.5359 | 0 |
| is_structural_domain_or_motif_of_gene_product | 0.0013 | 0.1374 | 0.0244 | 0 |
| is_location_of_biological_process | 0.0011 | 0.1118 | 3.6119 | 0 |
| chemical_or_drug_initiates_biological_process | 0.0009 | 0.0927 | 0.4783 | 0 |
| tissue_is_expression_site_of_gene_product | 0.0008 | 0.0828 | 0.6599 | 0 |
| gene_encodes_gene_product | 0.0000 | 0.0000 | 0.1414 | 0 |
| concept_in_subset | 0.0000 | 0.0000 | 0.0506 | 0 |
| subset_includes_concept | 0.0000 | 0.0000 | 0.0655 | 0 |
| special_category_includes_neoplasm | 0.0000 | 0.0000 | 0.0227 | 1 |
| gene_product_affected_by_chemical_or_drug | 0.0000 | 0.0000 | 0.0218 | 0 |
| gene_product_is_biomarker_of | 0.0000 | 0.0000 | 0.0367 | 0 |
| mechanism_of_action_of | -0.0001 | -0.0135 | 0.0751 | 0 |
| gene_product_has_associated_anatomy | -0.0003 | -0.0324 | 1.0300 | 0 |
| gene_product_plays_role_in_biological_process | -0.0004 | -0.0384 | 1.1539 | 0 |
| gene_product_has_organism_source | -0.0009 | -0.0889 | 0.5019 | 0 |
| is_organism_source_of_gene_product | -0.0014 | -0.1397 | 0.6232 | 0 |
| excised_anatomy_has_procedure | -0.0019 | -0.2246 | 0.3169 | 0 |
| organism_has_gene | -0.0028 | -0.2777 | 0.3195 | 0 |
| biological_process_is_part_of_process | -0.0029 | -0.3327 | 0.3160 | 0 |
| has_therapeutic_class | -0.0044 | -0.4547 | 0.2880 | 0 |
| process_involves_gene | -0.0060 | -0.6060 | 0.1466 | 0 |
| process_includes_biological_process | -0.0056 | -0.6313 | 0.3500 | 0 |
| gene_product_encoded_by_gene | -0.0069 | -0.6944 | 0.1248 | 0 |
| disease_has_normal_tissue_origin | -0.0065 | -0.7493 | 0.1693 | 0 |
| is_chemical_classification_of_gene_product | -0.0057 | -0.8755 | 0.0969 | 0 |
| gene_plays_role_in_process | -0.0103 | -1.0256 | 0.1711 | 0 |
| disease_has_normal_cell_origin | -0.0135 | -1.5474 | 0.5063 | 0 |
| disease_has_finding | -0.0111 | -1.7241 | 0.0794 | 0 |
| biomarker_type_includes_gene_product | -0.0189 | -1.9204 | 0.0244 | 0 |
| has_contraindicating_class | -0.0188 | -3.2581 | 0.0847 | 0 |
| disease_has_abnormal_cell | -0.0272 | -3.3843 | 0.2514 | 0 |
| induced_by | -0.0332 | -4.5455 | 0.0297 | 0 |
| is_not_primary_anatomic_site_of_disease | -0.0250 | -4.5926 | 0.0402 | 0 |
| process_initiates_biological_process | -0.0320 | -4.7075 | 0.0471 | 0 |
| product_component_of | -0.0313 | -4.8913 | 0.0122 | 0 |
| contraindicating_class_of | -0.0353 | -5.5789 | 0.0943 | 0 |
| gene_associated_with_disease | -0.0542 | -5.9891 | 0.0340 | 0 |
| procedure_has_completely_excised_anatomy | -0.0413 | -7.4286 | 0.0192 | 0 |
| biological_process_has_result_biological_process | -0.0617 | -7.7839 | 0.0253 | 0 |
| completely_excised_anatomy_has_procedure | -0.0627 | -11.8519 | 0.0166 | 0 |
| gene_mapped_to_disease | -0.1923 | -27.7778 | 0.0140 | 0 |
| disease_excludes_normal_cell_origin | -0.0464 | -34.7826 | 0.0340 | 1 |
