# Supplementary figures and images for: Large-Scale Biomedical Relation Extraction Across Diverse Relation Types: Model Development and Usability Study on COVID-19
Source: J Med Internet Res. 2023 Sep 20;25:e48115. doi: 10.2196/48115 (PMC10551783; doi:10.2196/48115)

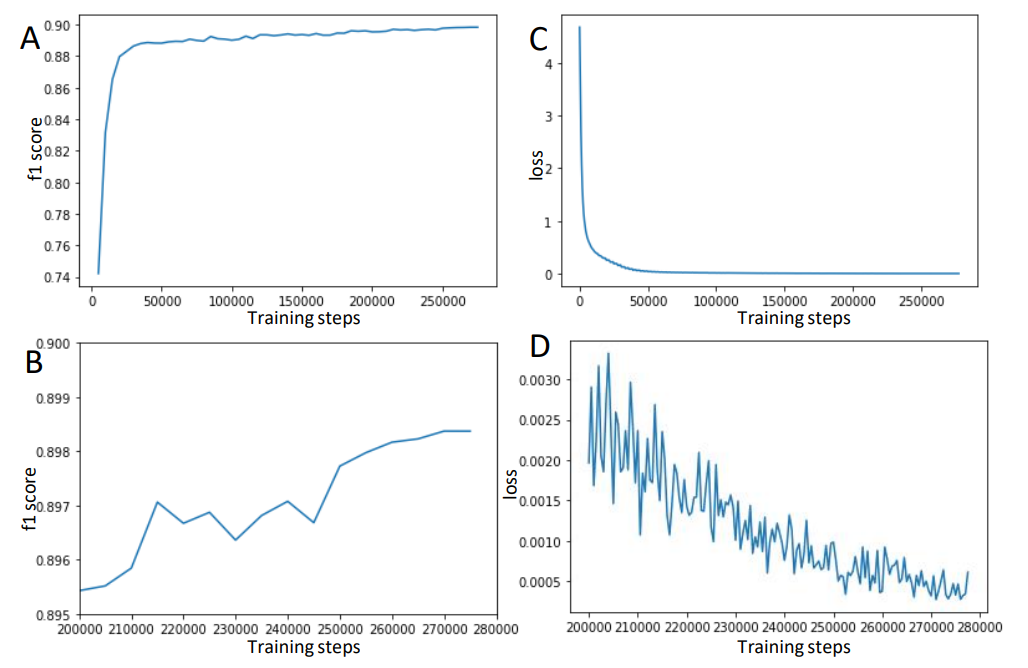


**Multimedia Appendix 6.** F1-score and loss trend for 100 epochs.

Supplement: Multimedia Appendix 6 [file jmir_v25i1e48115_app6.docx]

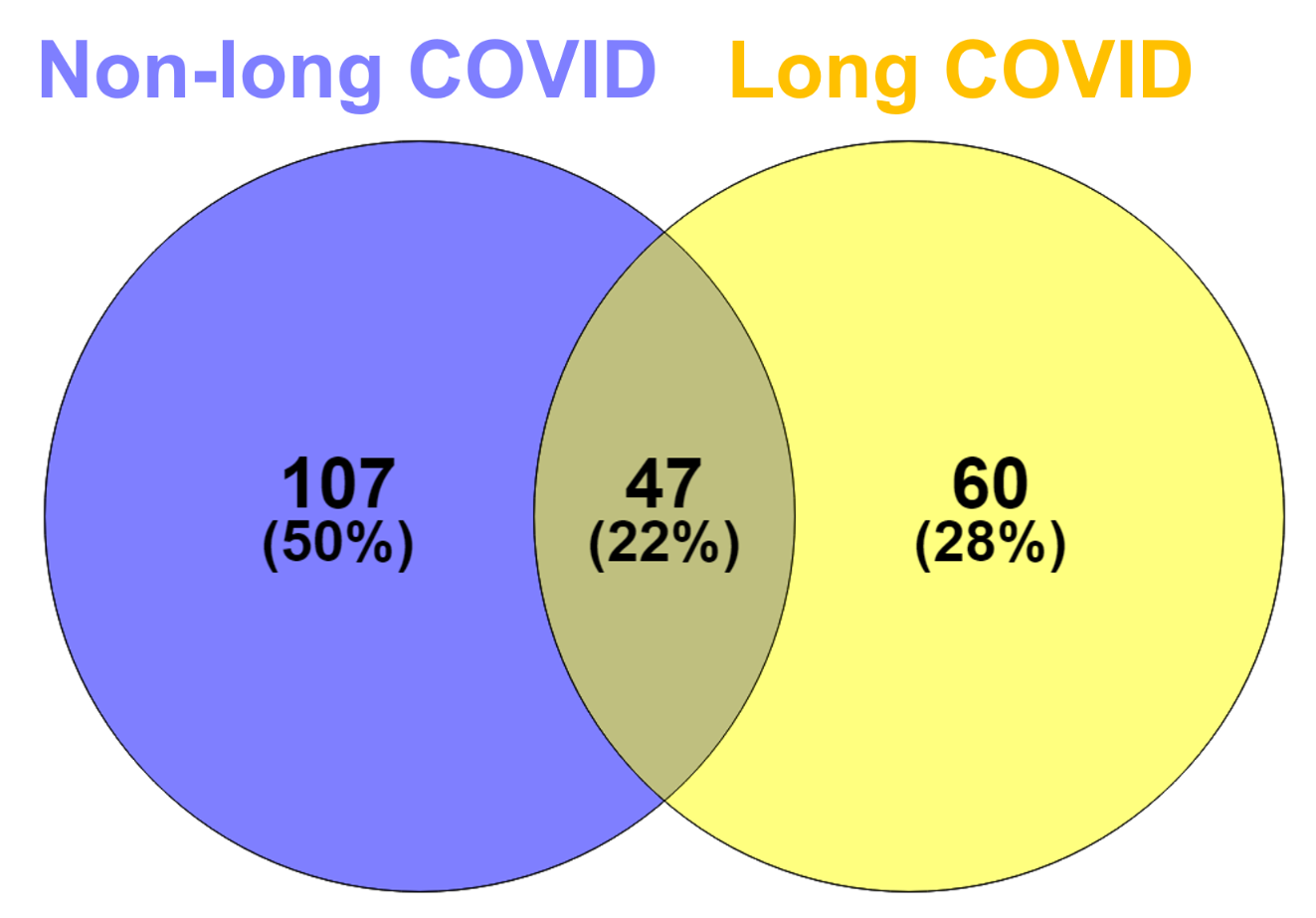


Multimedia Appendix 8. Venn diagram of drugs between non-long COVID and long COVID.

Supplement: Multimedia Appendix 8 [file jmir_v25i1e48115_app8.docx]
