# Supplementary material for: Large-Scale Biomedical Relation Extraction Across Diverse Relation Types: Model Development and Usability Study on COVID-19
Source: J Med Internet Res. 2023 Sep 20;25:e48115. doi: 10.2196/48115 (PMC10551783; doi:10.2196/48115)
Supplement: Multimedia Appendix 7 [file jmir_v25i1e48115_app7.docx]

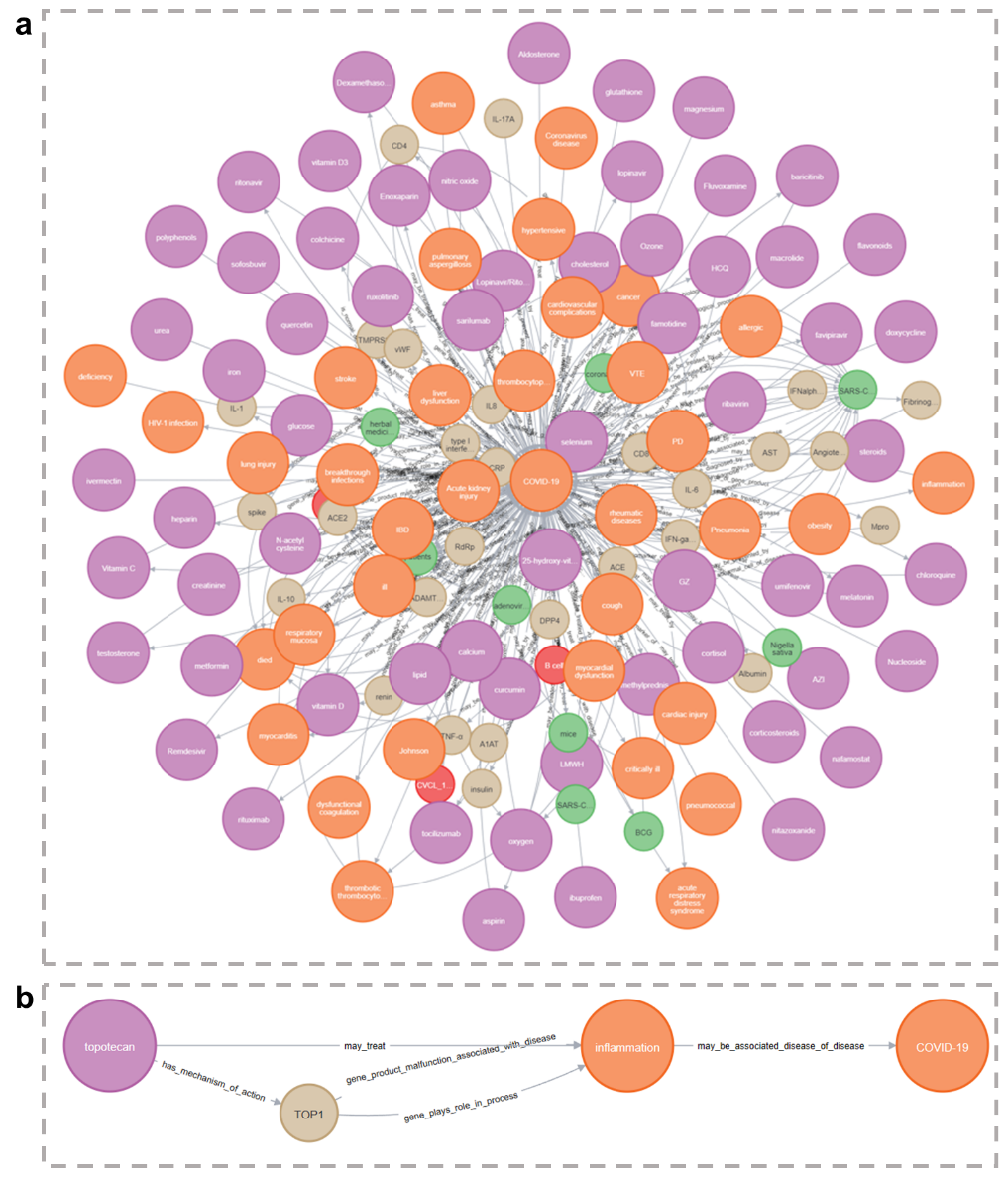


**Multimedia Appendix 7. Relation graph database visualization. (a)** Demonstration of COVID-19 relation graph database based on corpus from LitCovid. The orange nodes are diseases, the brown nodes are genes or proteins, the purple nodes are chemicals or drugs. **(b)** visualization of an example for potential drug to COVID-19 in relation graph database.
