# Supplementary material for: Large-Scale Biomedical Relation Extraction Across Diverse Relation Types: Model Development and Usability Study on COVID-19
Source: J Med Internet Res. 2023 Sep 20;25:e48115. doi: 10.2196/48115 (PMC10551783; doi:10.2196/48115)
Supplement: Multimedia Appendix 10 [file jmir_v25i1e48115_app10.docx]

**Multimedia Appendix 10.** Top 100 misprediction types by percentage.

| Gold standard | Prediction | % of total errors |
| --- | --- | --- |
| anatomic_structure_is_physical_part_of | has_physical_part_of_anatomic_structure | 7.03 |
| has_physical_part_of_anatomic_structure | anatomic_structure_is_physical_part_of | 5.95 |
| nichd_parent_of | not_a_relation | 4.88 |
| not_a_relation | nichd_parent_of | 4.57 |
| not_a_relation | has_nichd_parent | 4.44 |
| has_nichd_parent | not_a_relation | 4.05 |
| nichd_parent_of | has_nichd_parent | 3.31 |
| has_nichd_parent | nichd_parent_of | 2.91 |
| is_associated_anatomic_site_of | is_primary_anatomic_site_of_disease | 2.34 |
| is_primary_anatomic_site_of_disease | is_associated_anatomic_site_of | 2.24 |
| not_a_relation | chemical_structure_of | 2.20 |
| disease_has_primary_anatomic_site | disease_has_associated_anatomic_site | 2.07 |
| disease_has_associated_anatomic_site | disease_has_primary_anatomic_site | 2.04 |
| chemical_structure_of | not_a_relation | 1.83 |
| not_a_relation | is_biochemical_function_of_gene_product | 1.63 |
| is_biochemical_function_of_gene_product | not_a_relation | 1.57 |
| gene_product_has_biochemical_function | not_a_relation | 1.39 |
| contraindicated_with_disease | may_treat | 1.37 |
| may_prevent | may_treat | 1.32 |
| has_contraindicated_drug | may_be_treated_by | 1.11 |
| not_a_relation | gene_product_has_biochemical_function | 1.10 |
| may_be_prevented_by | may_be_treated_by | 0.85 |
| has_chemical_structure | not_a_relation | 0.77 |
| may_be_treated_by | has_contraindicated_drug | 0.75 |
| may_treat | may_prevent | 0.74 |
| not_a_relation | has_chemical_structure | 0.74 |
| is_normal_cell_origin_of_disease | is_abnormal_cell_of_disease | 0.67 |
| may_treat | contraindicated_with_disease | 0.66 |
| is_abnormal_cell_of_disease | is_normal_cell_origin_of_disease | 0.63 |
| disease_has_normal_cell_origin | disease_has_abnormal_cell | 0.63 |
| not_a_relation | has_ingredient | 0.59 |
| is_location_of_anatomic_structure | has_physical_part_of_anatomic_structure | 0.58 |
| has_physical_part_of_anatomic_structure | is_location_of_anatomic_structure | 0.56 |
| may_be_treated_by | may_be_prevented_by | 0.52 |
| cdrh_parent_of | not_a_relation | 0.48 |
| has_free_acid_or_base_form | not_a_relation | 0.48 |
| anatomic_structure_has_location | anatomic_structure_is_physical_part_of | 0.47 |
| disease_has_abnormal_cell | disease_has_normal_cell_origin | 0.47 |
| contraindicated_with_disease | may_prevent | 0.45 |
| has_cdrh_parent | has_nichd_parent | 0.42 |
| anatomic_structure_is_physical_part_of | anatomic_structure_has_location | 0.41 |
| procedure_has_target_anatomy | procedure_has_excised_anatomy | 0.41 |
| not_a_relation | has_tradename | 0.40 |
| partially_excised_anatomy_has_procedure | excised_anatomy_has_procedure | 0.38 |
| has_cdrh_parent | not_a_relation | 0.37 |
| target_anatomy_has_procedure | excised_anatomy_has_procedure | 0.36 |
| tradename_of | has_tradename | 0.36 |
| biological_process_has_associated_location | biological_process_has_result_anatomy | 0.35 |
| has_ingredient | not_a_relation | 0.33 |
| anatomic_structure_is_physical_part_of | is_location_of_anatomic_structure | 0.33 |
| anatomic_structure_has_location | has_physical_part_of_anatomic_structure | 0.32 |
| anatomic_structure_has_location | is_location_of_anatomic_structure | 0.31 |
| has_cdrh_parent | cdrh_parent_of | 0.31 |
| is_chemical_classification_of_gene_product | not_a_relation | 0.29 |
| has_salt_form | not_a_relation | 0.29 |
| is_location_of_anatomic_structure | anatomic_structure_is_physical_part_of | 0.29 |
| gene_product_has_chemical_classification | not_a_relation | 0.28 |
| anatomy_originated_from_biological_process | is_location_of_biological_process | 0.28 |
| process_includes_biological_process | biological_process_is_part_of_process | 0.28 |
| is_location_of_biological_process | anatomy_originated_from_biological_process | 0.28 |
| not_a_relation | has_cdrh_parent | 0.28 |
| is_location_of_anatomic_structure | anatomic_structure_has_location | 0.27 |
| not_a_relation | cdrh_parent_of | 0.27 |
| has_contraindicated_drug | may_be_prevented_by | 0.27 |
| cdrh_parent_of | nichd_parent_of | 0.27 |
| not_a_relation | gene_product_has_chemical_classification | 0.27 |
| not_a_relation | is_chemical_classification_of_gene_product | 0.26 |
| disease_excludes_normal_cell_origin | disease_has_normal_cell_origin | 0.26 |
| excised_anatomy_has_procedure | partially_excised_anatomy_has_procedure | 0.25 |
| procedure_has_partially_excised_anatomy | procedure_has_excised_anatomy | 0.25 |
| not_a_relation | has_active_metabolites | 0.24 |
| biological_process_has_result_anatomy | biological_process_has_associated_location | 0.24 |
| may_be_prevented_by | has_contraindicated_drug | 0.24 |
| has_tradename | not_a_relation | 0.24 |
| conceptual_part_of | has_conceptual_part | 0.23 |
| may_be_associated_disease_of_disease | disease_may_have_associated_disease | 0.23 |
| is_not_normal_cell_origin_of_disease | is_normal_cell_origin_of_disease | 0.23 |
| is_associated_anatomy_of_gene_product | tissue_is_expression_site_of_gene_product | 0.21 |
| may_prevent | contraindicated_with_disease | 0.20 |
| biological_process_is_part_of_process | process_includes_biological_process | 0.20 |
| disease_has_normal_tissue_origin | disease_has_primary_anatomic_site | 0.19 |
| has_conceptual_part | conceptual_part_of | 0.19 |
| contraindicating_class_of | not_a_relation | 0.19 |
| gene_product_has_biochemical_function | is_biochemical_function_of_gene_product | 0.19 |
| gene_product_has_associated_anatomy | gene_product_expressed_in_tissue | 0.19 |
| contraindicating_class_of | has_contraindicating_class | 0.19 |
| has_contraindicating_class | not_a_relation | 0.19 |
| not_a_relation | has_free_acid_or_base_form | 0.19 |
| not_a_relation | disease_may_have_associated_disease | 0.19 |
| gene_product_expressed_in_tissue | gene_product_has_associated_anatomy | 0.19 |
| active_metabolites_of | has_active_metabolites | 0.19 |
| procedure_has_excised_anatomy | procedure_has_target_anatomy | 0.19 |
| may_be_diagnosed_by | may_be_treated_by | 0.18 |
| is_biochemical_function_of_gene_product | gene_product_has_biochemical_function | 0.17 |
| procedure_has_excised_anatomy | procedure_has_partially_excised_anatomy | 0.17 |
| has_contraindicating_class | contraindicating_class_of | 0.16 |
| has_active_metabolites | not_a_relation | 0.16 |
| tissue_is_expression_site_of_gene_product | is_associated_anatomy_of_gene_product | 0.16 |
| disease_has_primary_anatomic_site | disease_has_normal_tissue_origin | 0.16 |
| ingredient_of | not_a_relation | 0.15 |
